# Application of artificial intelligence in head and neck tumor segmentation: a comparative systematic review and meta-analysis between PET and PET/CT modalities

**DOI:** 10.1186/s12885-025-14881-8

**Published:** 2025-10-27

**Authors:** Hamed Hajimokhtari, Tina Soleymanpourshamsi, Leila Rostamian, Ailar Yousefbeigi, Soheil Jafari, Asal Rezaeiyazdi, Mohammadjavad Askari, Maryam Khalilian, Parsa Vafaei, Mahla Esfahaniani, Gianrico Spagnuolo, Shirin Shahnaseri, Parisa Soltani

**Affiliations:** 1 Islamic Azad University of Isfahan, Isfahan, Iran; 2 Tehran University of Medical Sciences, Tehran, Iran; 3 University of California, Los Angeles, Los Angeles, USA; 4 Isfahan University of Medical Sciences, Isfahan, Iran; 5 Golestan University of Medical Sciences, Gorgan, Iran; 6 University of Naples Federico II, Naples, Italy; 7 Meharry Medical College, Nashville, USA

**Keywords:** Artificial intelligence, PET/CT, Tumor segmentation, Head and neck cancer, Deep learning, Radiomics

## Abstract

**Background:**

For the effective treatment planning of head and neck cancers, precise tumor segmentation is vital. The combination of artificial intelligence (AI) technology with imaging systems like positron emission tomography (PET) and PET/ computed tomography (PET/CT) has made attempts to automate these processes. Despite these attempts, the usefulness of AI segmentation with PET imaging compared to PET/CT still lacks clarity.

**Methods:**

A comprehensive search was performed on Scopus, Embase, PubMed, Cochrane, Web of Science, and Google Scholar for studies published before Dec 2024, with an update in March 2025. Included studies utilized AI algorithms to segment head and neck tumors via PET or PET/CT and provided quantitative performance measures. Pooled estimates of Dice Similarity Coefficient (DSC) sensitivity, precision, and Hausdorff Distance (HD95) were calculated using a random-effects model. Also, sensitivity analyses were performed to find the potential source of heterogeneity. Additionally, subgroup analyses were conducted for overall and primary tumor segmentation. Publication bias was assessed using weighted Egger’s test, followed by presentation of funnel plots for different metrics. Risk of bias (RoB) was evaluated using the QUADAS-C tool. Also, CLAIM was used to assess methodological quality and robustness of the included studies.

**Results:**

Eleven studies were included. All included studies were rated as having a low risk of bias. Also, CLAIM scores showed a high methodological quality in the studies. There was a significant difference between PET/CT and PET-only modalities. Pooled effectiveness metrics showed improvement in their respective DSC of 0.05 (95% CI 0.03–0.07), sensitivity by 0.04, and precision by 0.05, and HD95 decreased by approximately 3 mm. There was low heterogeneity for most metrics except HD95, which showed a high heterogeneity (I^2^ = 75%) and sensitivity, which showed a moderate heterogeneity (60.79%). Sensitivity analyses showed that leaving out the study by Dong et al. made the mean difference in HD95 smaller (from − 3.22 to − 1.82), but the result was still not statistically significant. When we did more sensitivity analysis by excluding SD-imputed studies, we found that the pooled effect sizes across all performance metrics did not change in direction or significance. We did subgroup analyses based on task type (overall vs. primary tumor segmentation) and modality comparison, and we found that all of the key metrics (Dice, Hausdorff Distance, Precision, Sensitivity) showed the same results, with no significant differences between the subgroups.

**Conclusions:**

The performance of AI-assisted segmentation using PET/CT is greater than that of PET-only in neck and head tumors. These results justify the clinical use of AI-based PET/CT imaging beyond contouring due to its automation potential and highlight the importance of unified datasets alongside distributed learning systems that improve the applicability and consistency of clinical workflows.

**Trial registration number:**

The study protocol was registered at PROSPERO [CRD42024614436].

**Supplementary Information:**

The online version contains supplementary material available at 10.1186/s12885-025-14881-8.

## Background

Head and neck cancer (HNC) is a common worldwide group of biologically distinct malignancies with complex anatomical presentation and high case fatality rates, demanding accurate staging, diagnosis, and treatment planning [1]. Head and neck cancers encompass a diverse group of malignancies that arise from various anatomical sub-regions, including the oral cavity, oropharynx, nasopharynx, hypopharynx, and larynx [2]. According to the Global Cancer Statistics 2022, head and neck cancers accounted for approximately 891,000 new cases and 458,000 deaths worldwide. These cancers comprised about 4.5% of all newly diagnosed cancer cases and 4.7% of global cancer-related deaths [3].

Positron emission tomography (PET) and computed tomography (CT) imaging modalities are now essential parts of this process, and they have the potential to generate functional and anatomical data [1, 4]. PET/CT enhances tumor segmentation through its combination of metabolic and anatomic data and is especially significant in gross tumor volume (GTV) delineation before radiation therapy [5, 6]. Every imaging modality has unique challenges and limitations in diagnosing HNC. With CT scans, tumor-lymph node metastases tend to obscure anatomical features critical to tissue evaluation during imaging, reducing precision [7]. PET scans face challenges in accurately identifying smaller tumors containing residual disease and post-treatment inflammation, which could result in inaccurate positive interpretations [8]. Additionally, in the PET/CT modality, the lack of consistent methods for defining the cutoff of metabolically active tumor regions due to interference from inflammatory uptake is a limitation [9]. However, PET/CT fusion imaging can compensate for the limitations of each modality by combining the high metabolic sensitivity of PET with the anatomical resolution of CT, thereby improving the delineation of tumor boundaries [10].

Precise segmentation of head and neck tumors provides important details regarding tumor size, location, and shape, which directly affects their radiotherapy and the patient’s prognosis. Hence, precise segmentation forms an integral part of treatment planning [11]. Accurate segmentation is crucial in radiotherapy planning, as errors in tumor boundary delineation may lead to insufficient tumor coverage or unnecessary irradiation of healthy tissue, ultimately affecting treatment outcomes [12]. Manual segmentation of tumors on PET or PET/CT scans is a time-consuming task prone to inter-observer and intra-observer variation, which inevitably compromises the reliability of treatment planning [13, 14]. Deep learning-based auto-segmentation processes have emerged as effective alternatives that can help reduce the workload without losing accuracy in tumor margin delineation [15, 16]. However, ambiguities such as unclear tumor boundaries, heterogeneity of gray-level values, and partial volume effect limit segmentation accuracy, especially in PET images [16, 17].

These gaps have motivated research on newer hybrid approaches using convolutional neural networks (CNNs) and uncertainty estimation methods for merging anatomical (CT) and functional (PET) data [13, 14]. In recent years, artificial, intelligence (AI) model, particularly convolutional neural networks (CNNs) and attention-based architectures, have shown promising capabilities in medical image segmentation. These models can automate and standardize tumor delineation, which is crucial for improving diagnostic precision and optimizing radiotherapy planning [18]. Precise tumor delineation in PET and PET/CT imaging is particularly challenging due to the low spatial resolution of PET and the heterogeneous nature of tumors in the head and neck region [19, 20]. While CT scans provide valuable anatomical context, they are often insufficient without additional information due to the CT’s inability to distinguish between tumors, inflammation, and necrosis [19, 21]. The combination of PET and CTs enables the use of metabolic and anatomical data, enhancing the localization and delineation of tumor boundaries. This is especially true when advanced deep learning architectures such as attention mechanisms and multimodal transformers are incorporated [20, 22]. Deep learning models using PET/CT have better generalizability and robustness when trained with data from multiple centers [5, 6].

Some research has proven that PET-CT fusion is more precise in tumor segmentation than a singular modality and significantly improves staging accuracy and treatment planning in head and neck cancers, especially in cases with locally advanced disease. It can detect metabolically active lesions not visible through conventional imaging [23–25]. Federated learning frameworks and cross-attention transformers have improved the generalizability of segmentation algorithms while tackling data privacy issues and institutional heterogeneity [20, 23]. The HECKTOR (Head and Neck Tumor Segmentation and Outcome Prediction in PET/CT images) challenge is an example of a benchmark initiative that has demonstrated the use of ensemble models and hybrid architectures [26, 27]. Held as part of MICCAI 2020 (Medical Image Computing and Computer-Assisted Intervention), the HECKTOR challenge was the first large-scale benchmark specifically focused on automatic segmentation of oropharyngeal tumors using PET/CT. It provided a high-quality multi-institutional dataset and standardized evaluation metrics to advance reproducible radiomics and AI development in head and neck cancer imaging [26].

However, it remains challenging to make conclusive judgments regarding the efficiency of PET-only and PET/CT-based segmentation models due to the discrepancies concerning the studies’ methodologies, datasets, and evaluation criteria [22, 28]. In addition, very few reviews [29, 30] have attempted to analyze these modalities more comprehensively, emphasizing artificial intelligence and deep learning in head and neck oncology. In 2022, Liu et al. [29] systematically reviewed the existing studies assessing the applications of deep learning in HNC prognosis. They categorized these applications into automatic segmentation, feature extraction, feature fusion, model building. In another study in 2024, Alabi et al. [30] performed a systematic review on the applications of AI-based radiomics for the management of HNC. Based on their review, the radiomics-based cancer diagnosis includes tumor staging, tumor grading, and classification of malignant and benign tumors, while the radiomics-based cancer prognosis includes prediction for treatment response, recurrence, metastasis, and survival. However, none of these reviews focused explicitly on PET and PET/CT and did not compare two modalities with a quantitative synthesis of results, including a meta-analysis.

Therefore, this review aims to systematically assess the current literature on PET-only and PET/CT-based AI segmentation of head and neck tumors, focusing on the identified methodological gaps and discussing their implications for practice. As far as we know, this is the first meta-analysis systematically investigating the AI segmentation performance of head and neck cancer using PET and PET/CT imaging modalities.

## Methods and materials

This study was performed in accordance with the guidelines for Preferred Reporting Items for a Systematic Review and Meta-analysis of Diagnostic Test Accuracy (PRISMA-DTA) [12]. The study protocol was registered at PROSPERO [CRD42024614436].

### Eligibility criteria

This systematic review addresses the following PICOS question: For HNC patients (population), does the AI-based PET/CT combination (intervention) compared with the AI-based PET-only (comparison) increase diagnostic and segmentation performance (outcome) in observational studies (study design)?

Studies that reported the following criteria were included:

1. Population: HNC patients, including nasopharyngeal carcinoma, HNC squamous cell carcinoma, Oropharyngeal cancers, thyroid cancers, parotid tumors, esophageal cancer, parathyroid adenoma, laryngeal cancer, and lymph node;
2. Intervention: AI-based PET/CT fusion diagnosis or segmentation of HNC;
3. Comparison: Comparing the performance of AI-based PET/CT segmentation with that of AI-based PET-only segmentation
4. Outcome: Diagnostic performance (accuracy, specificity, sensitivity, and precision) and segmentation accuracy (Dice Similarity Coefficient (DSC), Jaccard Index)
5. Study design: Observational studies

The following studies were excluded:

1. Studies that did not report sufficient details about training and testing datasets (medical imaging modality, dataset, etc.)
2. Studies that did not use AI for segmentation for both modalities
3. Studies that reported cancer outside the head and neck region
4. Studies on the central nervous system (including the brain, spinal cord, cerebrum, cerebellum, etc.)
5. Reviews, editorials, and conference papers without full data

To ensure sufficient statistical power and methodological reliability, studies were only included if they reported results on a minimum sample size of 30 patients. Furthermore, included studies were required to clearly specify a separation between training and test datasets to prevent data leakage and ensure the generalizability of model performance. Studies without explicit test set evaluation or lacking segmentation information for a distinct patient cohort were excluded.

### Information sources and search

An electronic search was conducted on Dec 1, 2024, and updated in March 2025 across the following six databases (with no time or language restrictions): Embase, PubMed, Cochrane, Scopus, Web of Science, and Google Scholar. Specific keywords and search queries were used for each database (Online Appendix 1). Also, Google Scholar, IADR abstracts, and ProQuest Theses and Dissertations databases were searched for grey literature. Additionally, the bibliographies of papers included in the study were manually checked for any additional studies.

### Study selection

Zotero 7 (Corporation for Digital Scholarship, Virginia, USA) was used to manage the citations. After removing duplicate papers, three independent reviewers (M.K. & A.R. & M.E.) screened the titles and abstracts. In the next stage, they screened full texts of eligible studies based on inclusion and exclusion criteria. A fourth independent reviewer resolved any disagreements. (P.V.)

### Data collection and extraction

Three reviewers (H.H. & T.S. & L.R.) independently extracted data from the included studies. A fourth reviewer (P.V.) revised the data collection to resolve discrepancies and disagreements. The following items were extracted: bibliographic details (author name and the publication year), country, cancer type assessed (accurate location or type), total sample size, dataset, the target of radiotherapy (primary tumor or lymph nodes), Imaging modalities, AI model used, AI training population (including training, validation, and test set), performance metrics assessed, and funding sources.

As mentioned before, All included studies’ funding sources were extracted and examined. Funding statements’ presence or absence was noted for transparency purposes and to assess the likelihood of sponsorship bias.

### Risk of bias and methodological quality assessment

The risk of bias was graded independently by three reviewers (H.H. & A.Y. & S.J.) using the quality assessment of diagnostic accuracy studies comparative (QUADAS-C) tool, and a fourth investigator (P.V.) resolved discrepancies between the reviewers [31]. The QUADAS-C tool, just like the QUADAS-2 tool, has four domains: data selection, index test, reference standard, and flow and timing. In addition, it has three domains for evaluating the applicability of a study: patient selection, index test, and reference standard. Studies were judged as low risk of bias in each domain if they provided sufficient methodological detail, used gold-standard reference tests consistently, and demonstrated appropriate data flow and timing.

Each domain was assessed and categorized as having a high, low, or unclear risk of bias. If limited information on the dataset was present, like unclear data split strategies and resulting data leakage, it was graded as a high risk of bias for the patient selection domain. In assessing the index test, studies were considered at high risk of bias if they lacked adequate reporting on test reproducibility, provided limited details on how the model was built, or failed to include robustness analyses. A high risk of bias was assigned for the reference standard domain when methods other than the gold standard—histopathological analysis—were used. Furthermore, if a study applied different reference standards within the same analysis or there was an inappropriate time gap between the index test and the reference standard, the flow and timing domain was also judged to be at high risk of bias. To evaluate concerns about applicability, we examined whether the dataset used, the deep learning model, its performance, and the annotation process aligned with the review’s objectives in each corresponding domain.

In addition to RoB assessment, the same four previous investigators assessed CLAIM (Checklist for Artificial Intelligence in Medical Imaging) to evaluate the methodological quality of the primary studies. The CLAIM is a reporting checklist created to guarantee transparency, reproducibility, and clinical relevance in research utilizing AI systems in medical imaging. It includes essential aspects such as data sufficiency, model construction and validation, performance analysis, explainability, and bias outlines. The AI-based segmentation models’ methodological master file and article reporting were checked in the CLAIM checklist, which offers a cohesive approach measuring the strength of parameters in the AI segmentation model.

### Synthesis

We synthesized data from the literature that included sensitivity, Precision, 95th percentile of the Hausdorff Distance (HD95), and Dice Similarity Coefficient (DSC). Only a handful of studies (n = 3) reported HD and Jaccard’s index, but sensitivity, precision, HD95, and DSC were stated in 10, 10, 9, and 14 studies, respectively. In many studies, recall is also called sensitivity or true positive rate (TPR). Additionally, precision is often referred to as positive predictive value interchangeably. With the provided approach below, missing metrics for each study were computed when adequate data were available in the reported data.

$$Dice = \frac{{\left( {Precision \times Sensitivity} \right) \times 2}}{Precision + Sensitivity}$$

Since one important study [32] reported the aggregated Dice similarity coefficient (DSCagg), and considering that DSCagg is generally higher than per-case DSC due to being calculated over the total volume, we estimated the per-case DSC by subtracting approximately 0.05 from the reported DSCagg, based on previous studies [33]. For studies that did not report standard deviations, we estimated SDs by multiplying the reported metric value by the mean SD-to-metric ratio derived from studies that provided both the metric and its SD, calculated separately for PET and PET/CT data. This approach for imputing missing standard deviations—based on the mean ratio of standard deviation to the reported metric across similar studies—is supported by Nakagawa et al. [34] recommended as a practical method when direct estimates are unavailable. It was applied to 8 out of 14 studies in the DSC metric, 5 out of 10 in sensitivity, 4 out of 9 in the HD95 metric, and 5 out of 10 in the precision metric. A sensitivity analysis, excluding these imputed cases, was also conducted to evaluate the impact of the approach used on the results.

### Statistical analyses

Meta-analyses were conducted using Stata version 17 (StataCorp, College Station, TX, USA). For each outcome, mean differences (MDs) with 95% confidence intervals (CIs) were calculated to compare the segmentation performance between PET/CT and PET-only modalities. Statistical heterogeneity across studies was assessed using Cochran’s Q test and the I^2^ statistic. A fixed-effects model with inverse-variance weighting was used when heterogeneity was low (I^2^ < 50% and *p* > 0.1), while a random-effects model using the restricted maximum likelihood (REML) estimator was applied in cases of moderate to high heterogeneity (I^2^ ≥ 50%).

Funnel plots were constructed to evaluate potential publication bias and small-study effects, plotting study precision (1/SE) against the effect size (MD). Asymmetry in the funnel plot was tested using Egger’s regression test.

Sensitivity analyses were performed in two ways to assess the robustness of pooled estimates. First, to examine the effect of imputing missing standard deviations (SDs), meta-analyses were repeated after excluding studies with estimated SDs (based on the mean SD-to-mean ratio of reporting studies). Second, for outcomes exhibiting moderate to high heterogeneity (I^2^ ≥ 50%), additional leave-one-out analyses were conducted to identify influential studies and assess their impact on the overall results.

Subgroup analyses were also performed, particularly stratified by segmentation target (e.g., primary tumor vs. overall volume), to explore possible sources of heterogeneity. All statistical tests were two-tailed, and *p*-values < 0.05 were considered statistically significant. The datasets used and analyzed during the current study are available from the corresponding author on reasonable request.

## Results

### Study selection

**Fig. 1:**
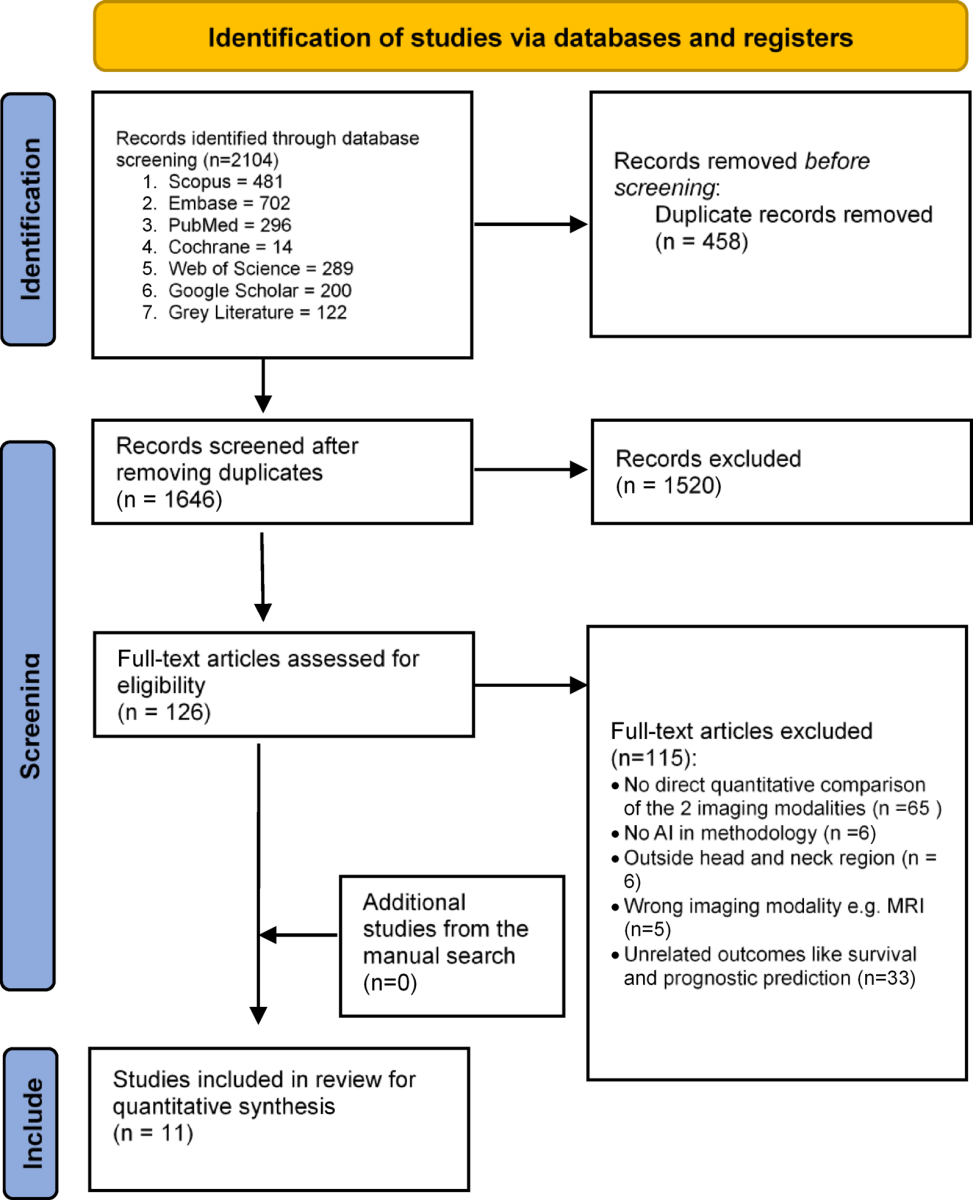
PRISMA flow diagram

Among the 1646 (After excluding duplicates) searched studies, 126 were included for further screening. After screening the full texts, 115 articles were excluded considering the exclusion criteria, and the remaining 11 articles were finally included (no additional studies were found with the manual search) (Fig. 1). No grey literature met the inclusion criteria due to the missing or incomplete data (sample size, standard deviation, etc.). Thus, only the peer-reviewed original articles were used to synthesize results. Online Appendix 2 presents the reasons for the exclusion of studies.

### Study characteristics

Table 1 summarizes individual studies. All the studies assessed PET/CT performance versus PET-only performance based on AI techniques in HNC segmentation. Studies reported five different types of malignancies: nasopharyngeal carcinoma (n = 4), oropharyngeal cancers (n = 7), hypopharyngeal cancers (n = 3), laryngeal cancers (n = 3), oral cavity cancers (n = 2), and squamous cell carcinoma of the head and neck (n = 2).

**Table 1:** Characteristics of the included studies

Author	Year	Design	Country	Cancer type	Sample size	Dataset	Target	Imaging Modality	AI Training Population			Metrics	Funding source reporting
									Training Set	Validation Set	Testing Set		
Dong et al. [24]	2024	Retrospective	USA	Oropharynx HNC	224 patients	Hecktor 2021	GTVt	CT, PET, CT/PET	170 patients	14 patients	40 patients	DSC HD95 Sensitivity Specificity FPR FNR	✓
Groendahl et al. [35]	2021	Retrospective	Norway	HNSCC (of the oral cavity, oropharynx, hypopharynx, and larynx)	197 patients	Oslo University Hospital and affiliated institutions in Norway	Overall	PET, CT, PET/CT	157 patients		40 patients		✓
Guo et al. [36]	2020	Retrospective	China & USA	HNSCC (Oropharynx, Larynx, Nasopharynx, Hypopharynx)	250 patients	TCIA (Cancer Imaging Archive)	Overall	CT, PET, CT/PET	140 patients	35 patients	75 patients	DSC HD95 MSD DC	✓
Huang et al. (ISA Net) [22]	2022	Retrospective	China	Head & Neck tumors (includes oropharynx)	275 patients (224 for Hecktor; 51 for STS)	STS (soft tissue sarcoma) & HECKTOR (head and neck tumor)	GTVt	CT, PET, CT/PET	80% of datasets		45 for Hecktor; 10 for STS	DSC HD ASSD	✓
Huang et al. (TG-NET) [37]	2022	Retrospective	China	Nasopharyngeal carcinoma	51 patients	Sun Yat-sen University Cancer Center,	GTVt	CT, PET, KI, and their combinations	40 patients		11 Patients	DSC HD95 Recall Precision VOE RVD	✓
Mahdi et al. [32]	2024	Retrospective	Saudi Arabia & Malaysia	HNC, especially in the oropharynx	883 patients	HECKTOR challenge at MICCAI from 9 different centers 2022	GTVt + GTVn + overall	FDG-PET/CT fusion PET CT	524 patients		359 Patients	DSCagg Jaccard ASD Precision recall VC	✓
Moe et al. [38]	2021	Retrospective	Norway	Head and Neck Cancer (SCC of the oral cavity, oropharynx, hypopharynx, and larynx)	197 patients	patients referred to curative chemoradiotherapy at Oslo University Hospital	GTVt + GTVn + overall	PET, CT, PET/CT	142 patients	15 patients	40 patients	Dice score, HD95, ASD, MSD, Coverage fraction, Sensitivity, PPV, Volume of true & false structures	✓
Oreiller et al. [26]	2022	Retrospective	Switzerland	Oropharyngeal tumor	254 patients	HECKTOR 2020 (MICCAI challenge dataset)	GTVt	PET, CT, PET/CT	201 patients		53 patients	DSC HD95 Precision recall SDSC	✓
Shiri et al. [28]	2023	Retrospective	Switzerland	HNC	328 patients	6 centers	GTVt	CT, PET, CT/PET fusion	70% of the whole dataset	10% of the entire dataset	20% of the entire dataset (66 patients)	Dice false negative false positive HD Jaccard	✓
Zhao et al. [39]	2019	Retrospective	China	Nasopharyngeal carcinoma	30 patients	PET Center of Southern Hospital & Guangzhou Military Region General Hospital	GTVt	PET, PET/CT	20 patients		10 patients	DSC PPV Sensitivity	✓
Zhao et al. [20]	2024	Retrospective	China	Nasopharyngeal carcinoma	51 patients	Hecktor + Sun Yat-sen University Cancer Center (self-collect)	GTVt	CT, PET, CT/PET	40 patients (Self-collect)		11 patients (self-collected) + 224 Patients Hecktor	DSC HD95 VOE RVD	✓

Additionally, among the included studies, nine major architecture types (16 with their variants) were used as AI techniques for segmentation. These architectures include U-net and its variants (n = 9), Denoising Diffusion Probabilistic Model (n = 2), Convolutional Neural Network (n = 2), DenseNet (n = 1), ISA-Net (n = 1), TG-Net (n = 1), Weighted Fusion Transformer (n = 1), U2-Net (n = 1), MMCA-Net (n = 1), Y-Net (n = 2), and Trans-Y-Net (n = 1).

Furthermore, studies targeted three main locations for segmentation: primary tumor segmentation (n = 9), lymph node segmentation (n = 2), and both GTVt and GTVn segmentation (n = 2).

Studies were categorized based on the imaging modality they employed. Among the studies, comparisons between PET/CT and PET-only modalities were common. In some studies, a further comparison, including CT or KI, was employed, too. Only one study did not use CT for comparison, in addition to PET and PET/CT (PET and PET/CT = 11, CT = 10).

The last but not least, all studies disclosed funding sources, and no industry-related sponsorship likely to influence the findings was noted.

### Risk of bias applicability & quality assessment

The QUADAS-C assessment showed that all 11 studies included in the review had a low risk of bias in the main areas of concern (Table 2). In Domain 1 (Patient Selection), all studies applied reasonable inclusion criteria, and the prevailing answers indicated minimal concern regarding selection bias. Regarding Domain 2 (Index Test), all studies independently reported the results, reducing the risk in this area. Similarly, the reference standards used in Domain 3 were deemed valid and independent of the interpretation of the index test, which provides further evidence on methodological soundness. There were no significant issues in Domain 4 (Flow and Timing), where all of the studies provided complete data for all patients, and there was temporal order between testing and the application of the reference standard. In general, the available evidence displayed sufficient methodological excellence, as indicated by all studies receiving a “low” risk of bias evaluation. This strengthens the reliability of their claimed diagnostic performance statistics. Online Appendix 3 contains all the details and complete scoring criteria.

**Table 2:**
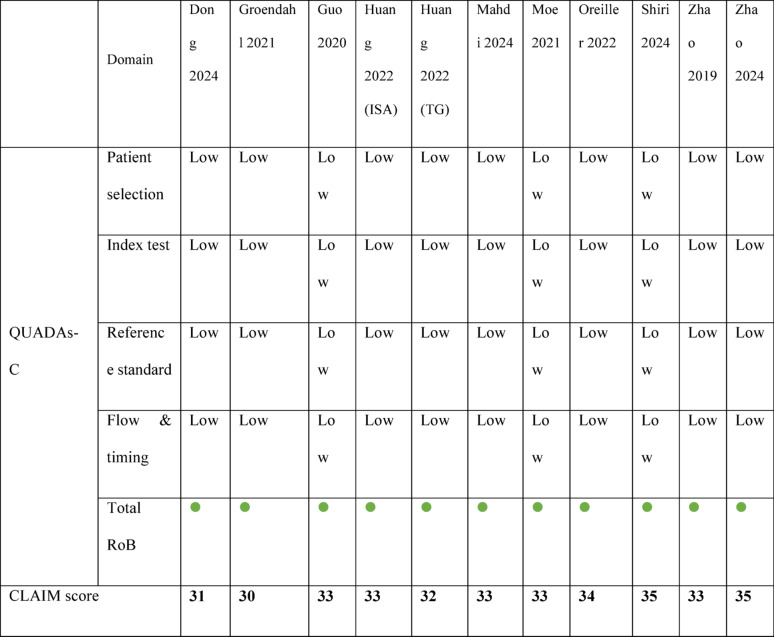
Details of the QUADAS-C and CLAIM scores of the included studies (Green light = Low risk of bias)

Bold values indicate the overall CLAIM score of each included study

The included studies were also evaluated using the CLAIM (Checklist for Artificial Intelligence in Medical Imaging) guideline. All studies demonstrated good compliance in performance, scoring between 30 and 35 out of a possible 42 points, averaging 32.9 points (Table 2). This low variability suggests that all the studies had high reporting quality. Although most of the studies captured the description of architecture, evaluation, and training/testing of the AI models, there were significant omissions, such as external validation and the absence of publicly available code or model weights. The study scores indicate that the included studies are methodologically sound. Online Appendix 4 contains comprehensive scoring details and full scoring breakdowns.

### Findings of the studies

#### Interpretation of meta-analytic results

This systematic review compared the efficiency of PET-only and PET/CT modalities in four different segmentation metrics: Dice Similarity Coefficient (DSC), Precision, Sensitivity, and Hausdorff Distance (HD95). The meta-analyses obviously showed advantages of PET/CT over PET-only in all segmentation metrics.

#### Dice similarity coefficient (DSC)

For Dice Similarity Coefficient (DSC), due to the absence of heterogeneity (I^2^ = 1.23%) and a non-significant Q statistic (*p* = 0.44), a fixed-effect model (Inverse-Variance method) was applied for pooling the results. The overall effect size was statistically significant (*p* < 0.001), indicating that the mean in the case group (PET/CT) was 0.05 higher than that of the control group (PET). The Egger’s test for publication bias was not significant (*p* = 0.958), suggesting no evidence of publication bias. Sensitivity analysis demonstrated that the exclusion of any single study did not affect the overall significance of the results. Subgroup analysis also revealed no heterogeneity in either group, and both subgroups showed statistically significant results. However, there was a slight difference in the magnitude of effect between the two groups, which was statistically significant (*p* = 0.04).

#### Hausdorff distance (HD)

For Hausdorff Distance (HD), due to the presence of heterogeneity (I^2^ = 90.72%) and a significant Q statistic (*p* < 0.001), a random-effects model (REML) was used to pool the results. The overall effect size was not statistically significant (*p* = 0.1), indicating no difference in the mean between the case group (PET/CT) and the control group (PET). Egger’s test for publication bias was not significant (*p* = 0.732), suggesting no evidence of publication bias. Sensitivity analysis showed that removing any individual study did not affect the overall result or its significance; however, the effect size changed more noticeably after excluding the study by Dong (from − 3.22 to − 1.82). Subgroup analysis revealed substantial and statistically significant heterogeneity in the overall group, whereas the primary tumor subgroup did not show significant heterogeneity. Nevertheless, the results were not statistically significant in either subgroup, and the difference between them was also not statistically significant (*p* = 0.15).

#### Precision

For precision, due to the absence of significant heterogeneity (I^2^ = 46.34%) and a non-significant Q statistic (*p* = 0.05), a fixed-effect model was used to pool the results. The overall effect size was statistically significant (*p* < 0.001), indicating that the mean in the case group (PET/CT) was 0.05 higher than that of the control group (PET). Egger’s test for publication bias was not significant (*p* = 0.732), suggesting no evidence of publication bias. Sensitivity analysis showed that excluding any individual study did not impact the overall results or their significance. Subgroup analysis indicated that there was no heterogeneity in either group, and both subgroups showed statistically significant results. Although there was a slight difference in the magnitude of the effect between the two groups, this difference was not statistically significant (*p* = 0.46).

#### Sensitivity

For sensitivity, due to the presence of heterogeneity (I^2^ = 60.79%) and a significant Q statistic (*p* = 0.01), a random-effects model was used to pool the results. The overall effect size was statistically significant (*p* = 0.01), indicating that the mean in the case group (PET/CT) was 0.05 higher than that of the control group (PET). Egger’s test for publication bias was not significant (*p* = 0.148), suggesting no evidence of publication bias. Sensitivity analysis showed that the exclusion of any individual study did not affect the overall result or its statistical significance. Subgroup analysis revealed that heterogeneity was significant in the primary tumor group but not in the overall group. Regarding the statistical significance of the effect size, the result was significant in the overall group but not in the primary tumor subgroup.

#### Publication bias

Publication bias was assessed using Egger’s test across all meta-analyses. None of the analyses demonstrated statistically significant evidence of publication bias, with *p*-values ranging from 0.148 (Sensitivity) to 0.958 (DSC). These findings suggest that the pooled estimates were unlikely to have been substantially influenced by selective publication of positive results. The lack of asymmetry in Egger’s test supports the reliability of the synthesized data, reinforcing the credibility of the overall meta-analytic conclusions.

### Sensitivity analyses

Sensitivity analyses were conducted to assess the robustness of the meta-analytic results through two approaches. First, a leave-one-out analysis was performed by sequentially excluding individual studies. Across all outcome measures (DSC, HD, Precision, and Sensitivity), the exclusion of any single study did not materially alter the statistical significance or direction of the overall effect sizes. An exception was observed for the HD metric, where removal of the study by Dong reduced the mean difference from − 3.22 to − 1.82, though the result remained statistically non-significant.

Second, an additional sensitivity analysis was performed by excluding all studies in which standard deviations were imputed using the mean SD-to-mean ratio method. The pooled estimates remained consistent in direction and magnitude, and the overall conclusions were unchanged. This further supports the stability and reliability of the meta-analytic findings, even under varying assumptions about missing data.

### Subgroup analyses

**Fig. 2:**
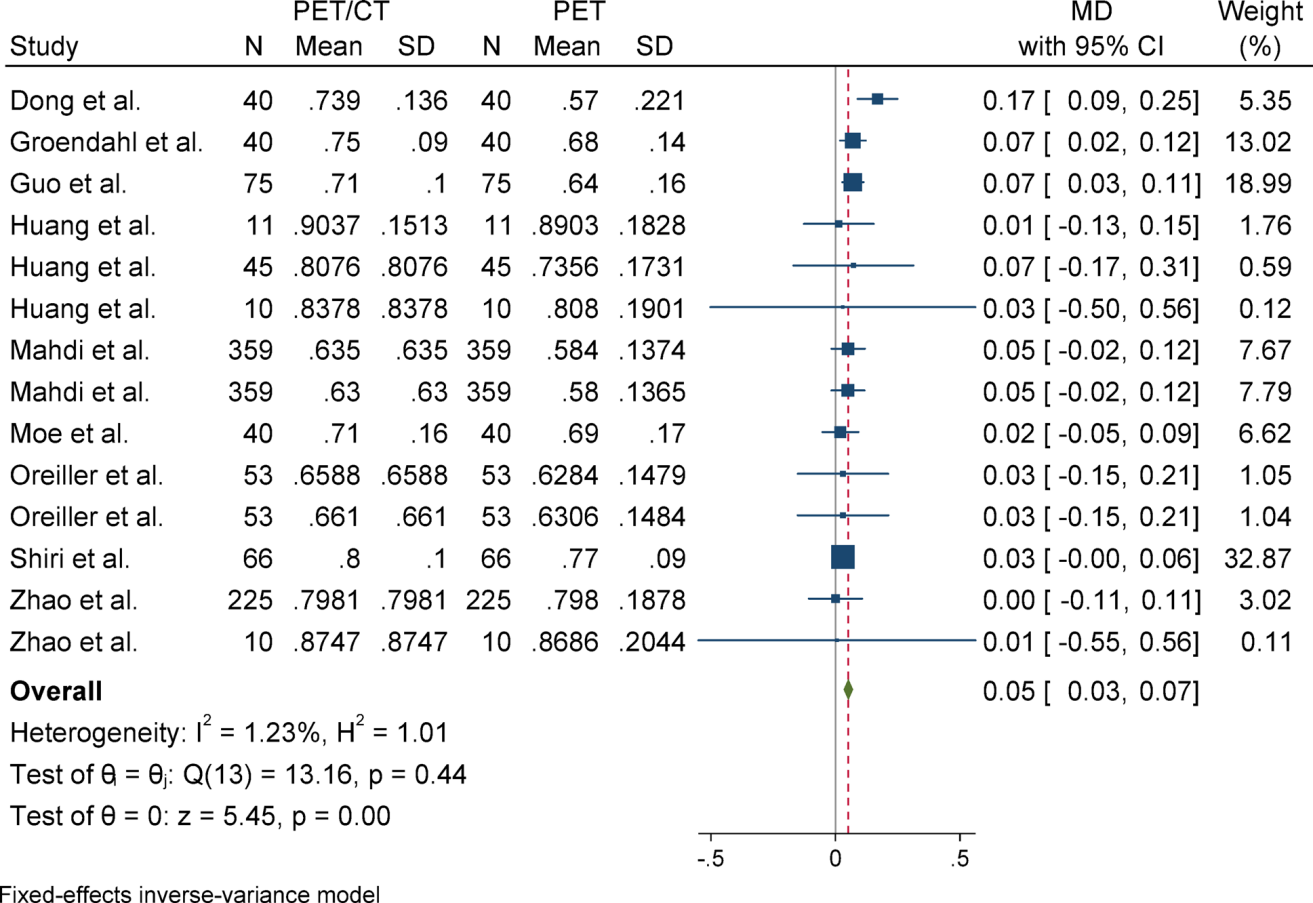
Forest plot for dice similarity coefficient (DSC); Forest plot shows the pooled mean difference (MD) of DSC between PET/CT and PET across 14 studies, favoring PET/CT (MD = 0.05, 95% CI 0.03 to 0.07) with low heterogeneity (I^2^ = 1.23%)

**Fig. 3:**
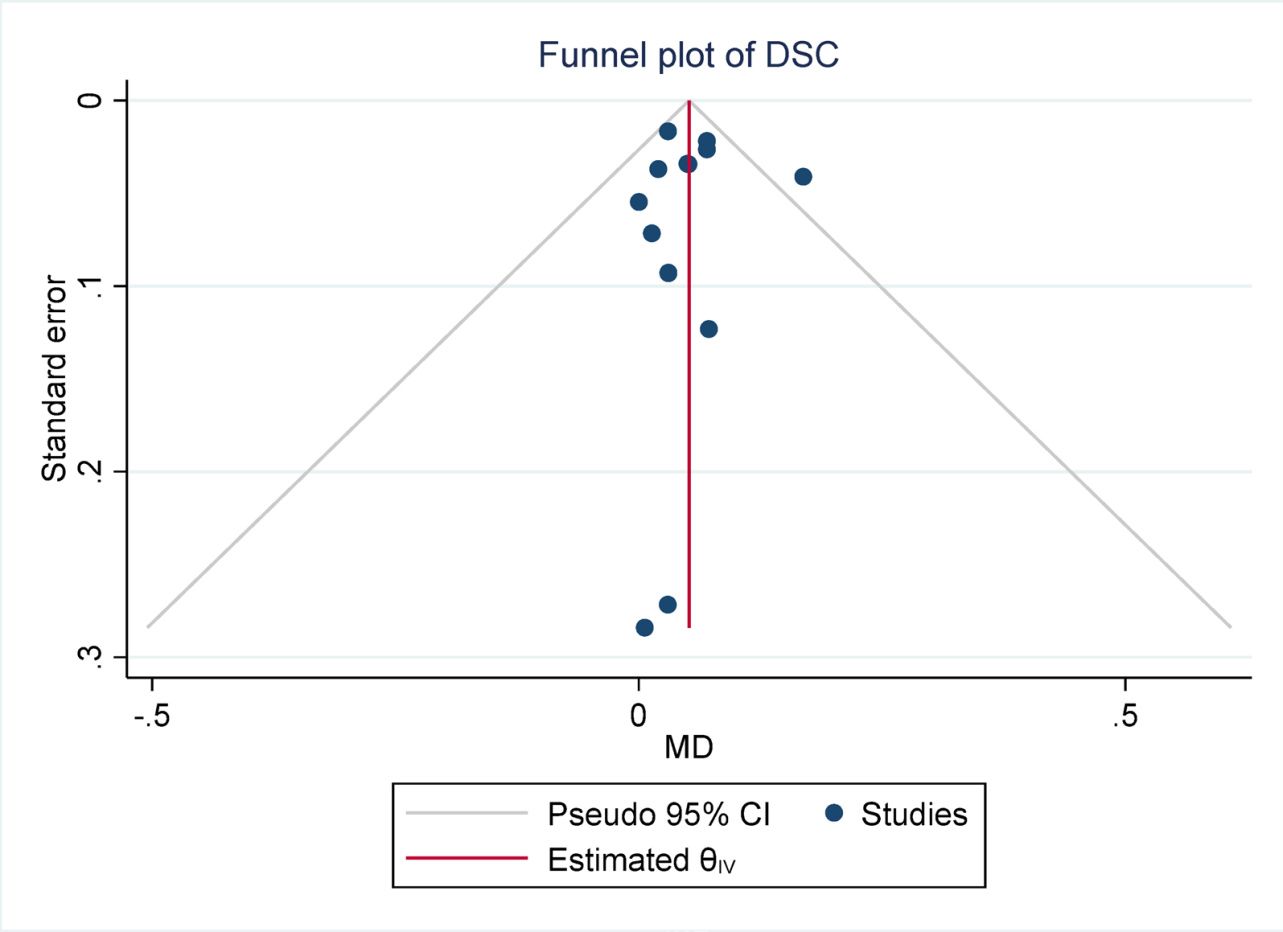
Funnel plot for dice similarity coefficient (DSC). The Egger test indicated no significant publication bias (*p* = 0.958)

**Fig. 4:**
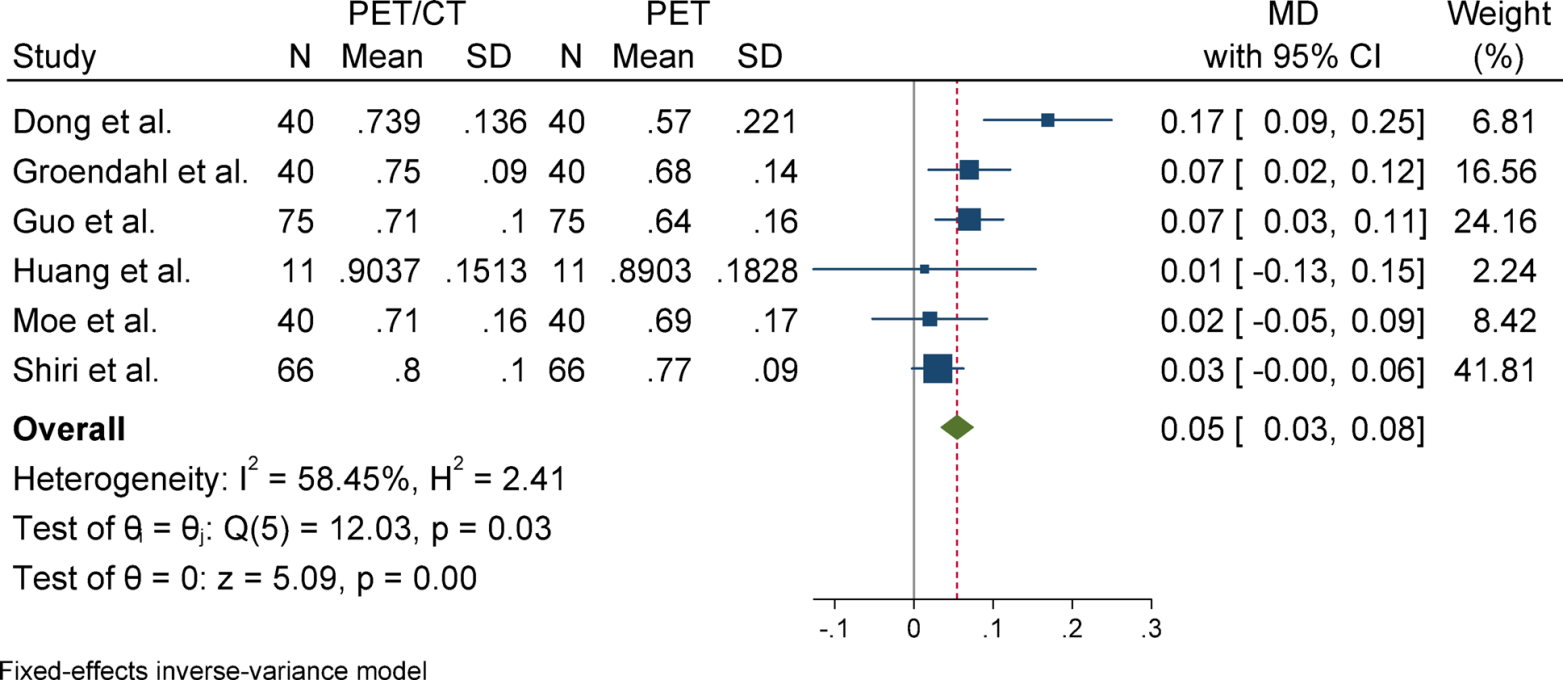
Forest plot for dice similarity coefficient (DSC) after removing SD-imputed studies The overall mean difference remained relatively stable compared to the initial analysis using imputed SDs, indicating the accuracy and robustness of the SD estimation method

**Fig. 5:**
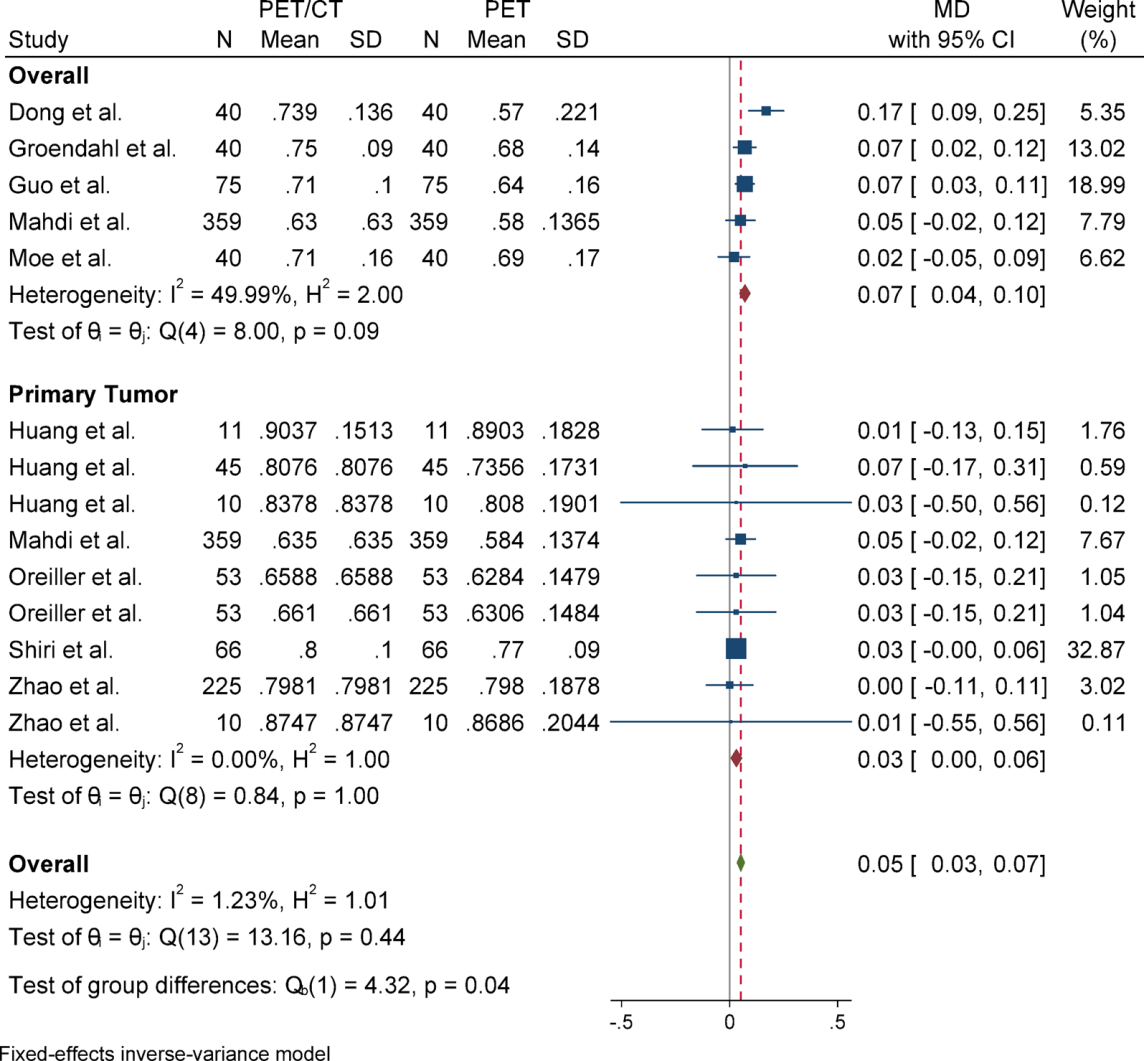
Subgroup analyses of DSC in terms of overall and primary tumor segmentation

**Fig. 6:**
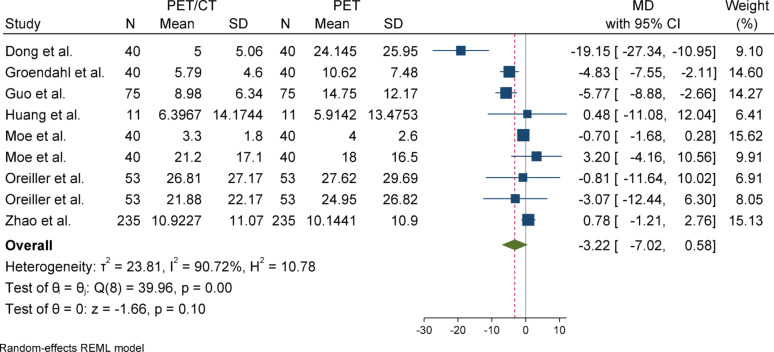
Forest plot for HD)Hausdorff distance(; Forest plot shows the pooled mean difference (MD) of HD between PET/CT and PET across 14 studies, favoring PET/CT (MD = − 3.22, 95% CI − 7.02 to 0.58) with high heterogeneity (I^2^ = 90.72%)

**Fig. 7:**
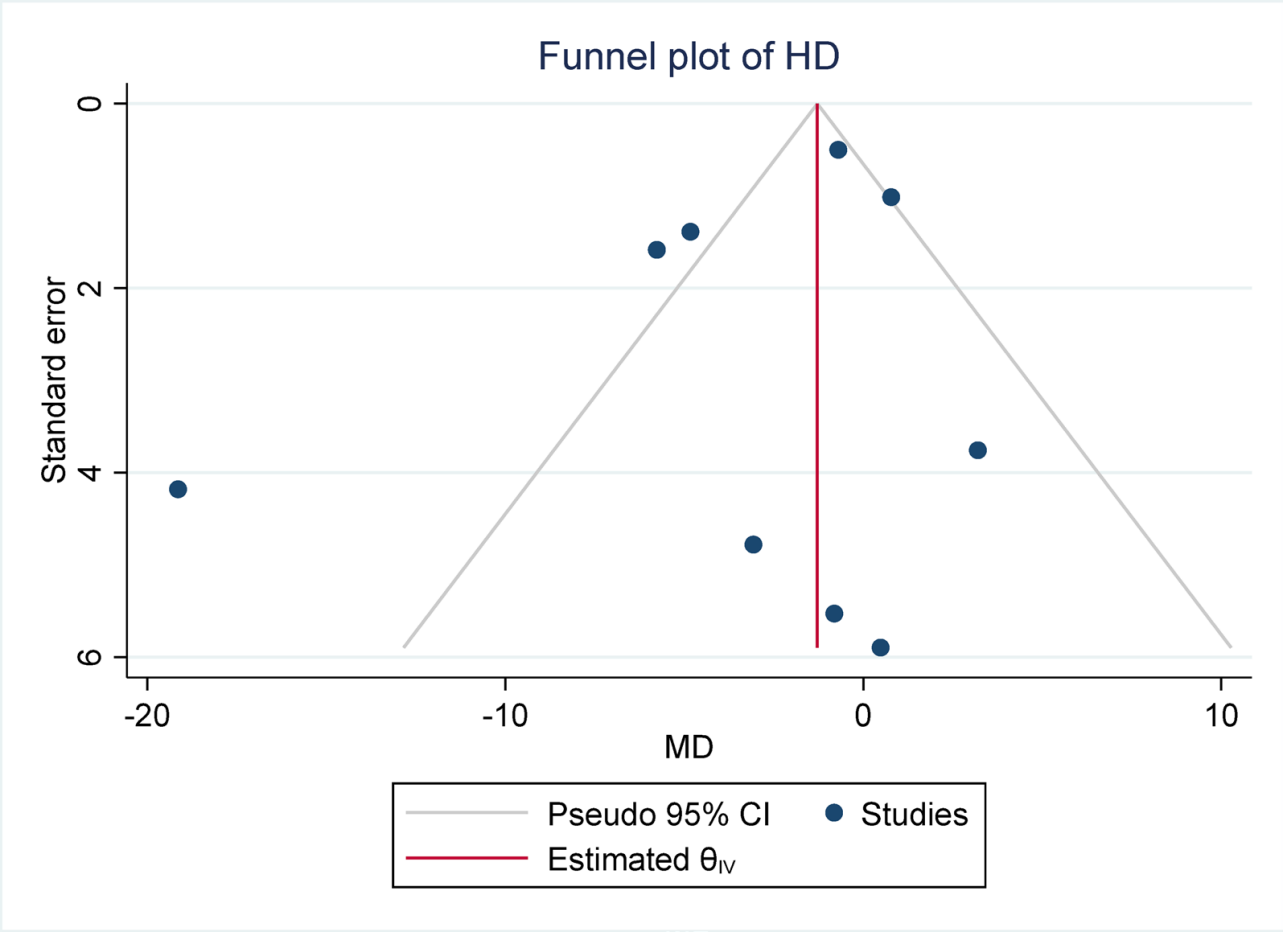
Funnel plot for HD (Hausdorff distance). The Egger test indicated no significant publication bias (*p* = 0.732)

**Fig. 8:**
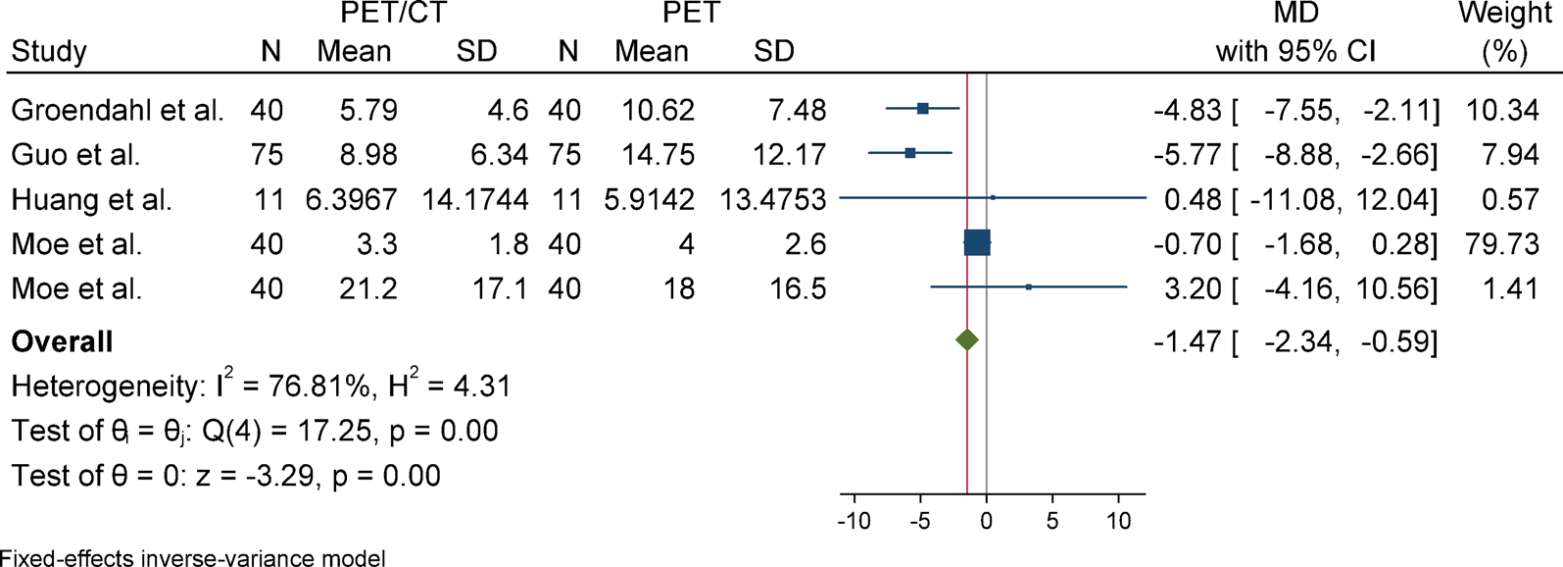
Forest plot for HD after removing SD-imputed studies “After excluding studies without reported standard deviations (SD), the mean difference in HD (Hausdorff Distance) decreased from, accompanied by a reduction in heterogeneity. These changes suggest improved consistency and reduced between-study variance after excluding incomplete data”

**Fig. 9:**
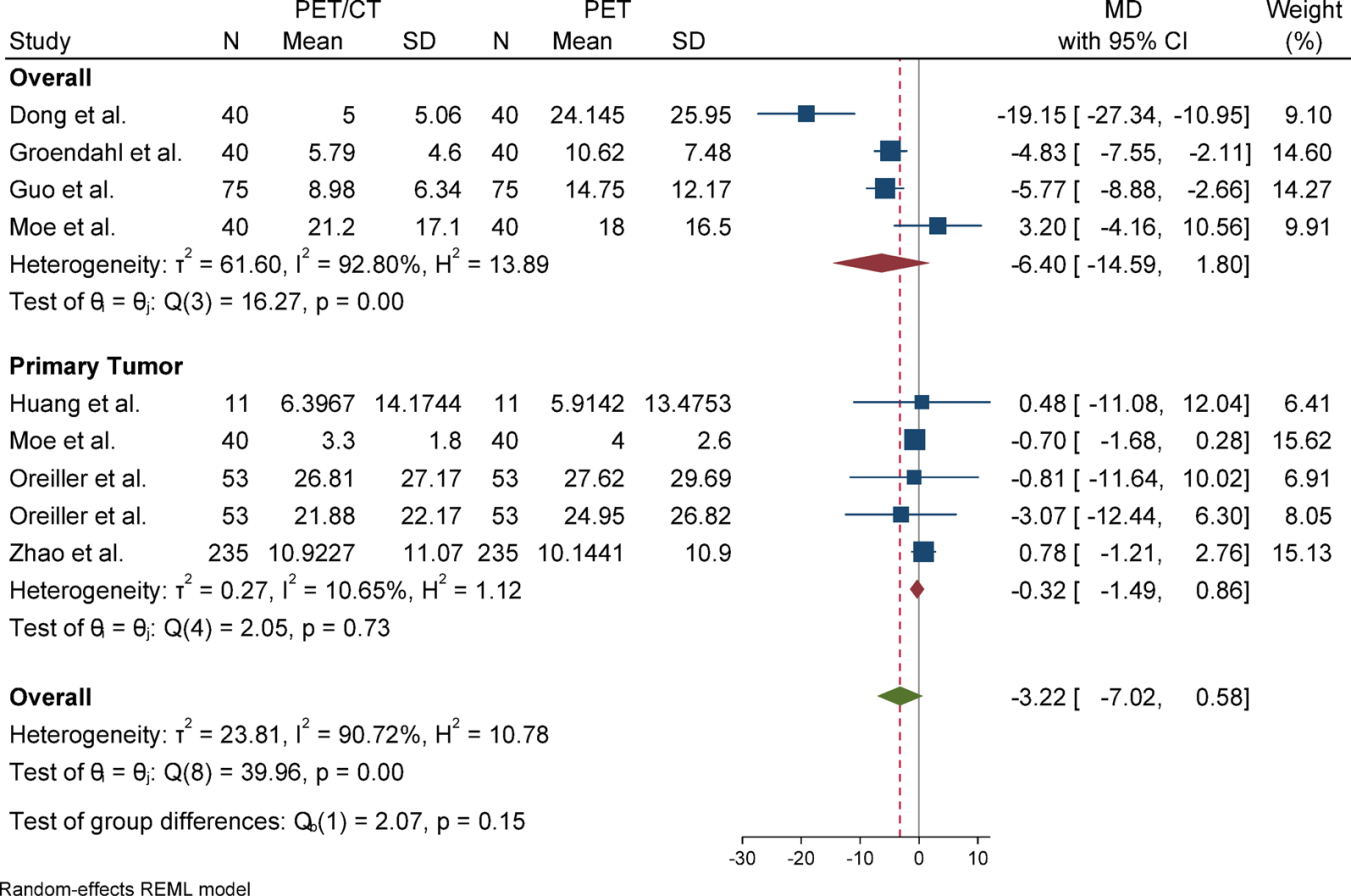
Subgroup analyses of HD in terms of overall and primary tumor segmentation

**Fig. 10:**
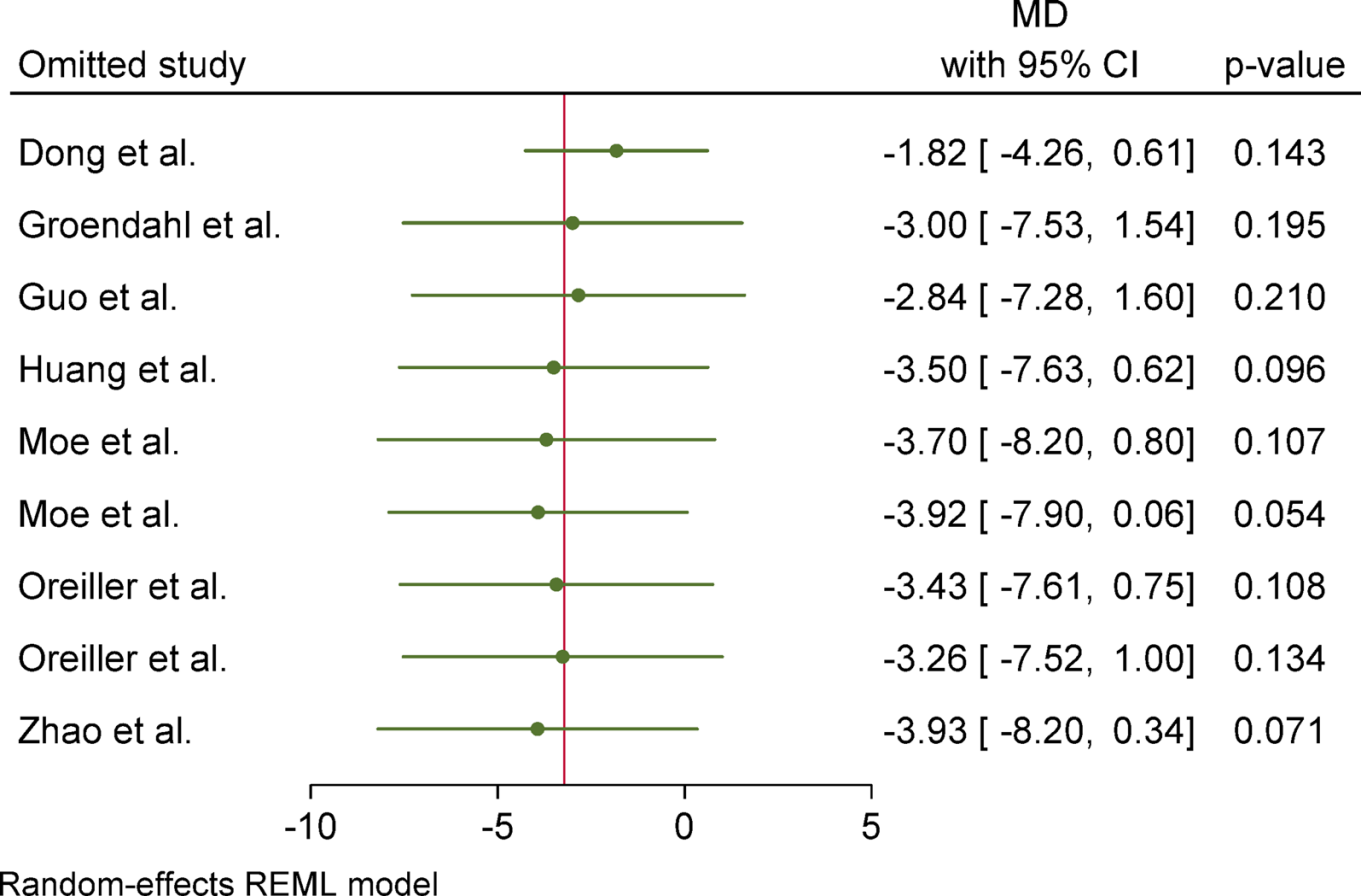
One-leave-out analysis for HD

**Fig. 11:**
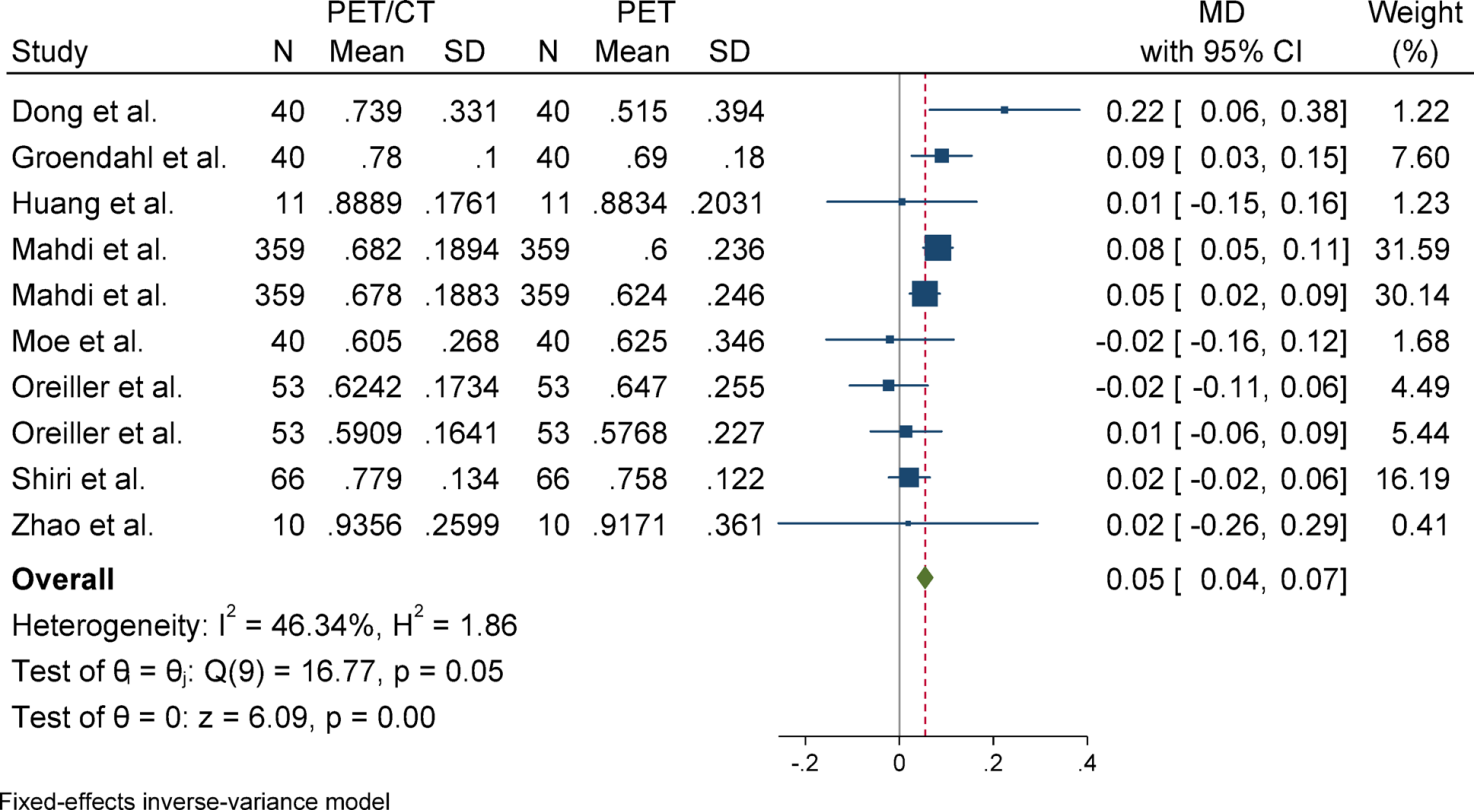
Forest plot for precision; Forest plot shows the pooled mean difference (MD) of precision between PET/CT and PET across 14 studies, favoring PET/CT (MD = 0.05, 95% CI 0.04 to 0.07) with low heterogeneity (I^2^ = 46.34%)

**Fig. 12:**
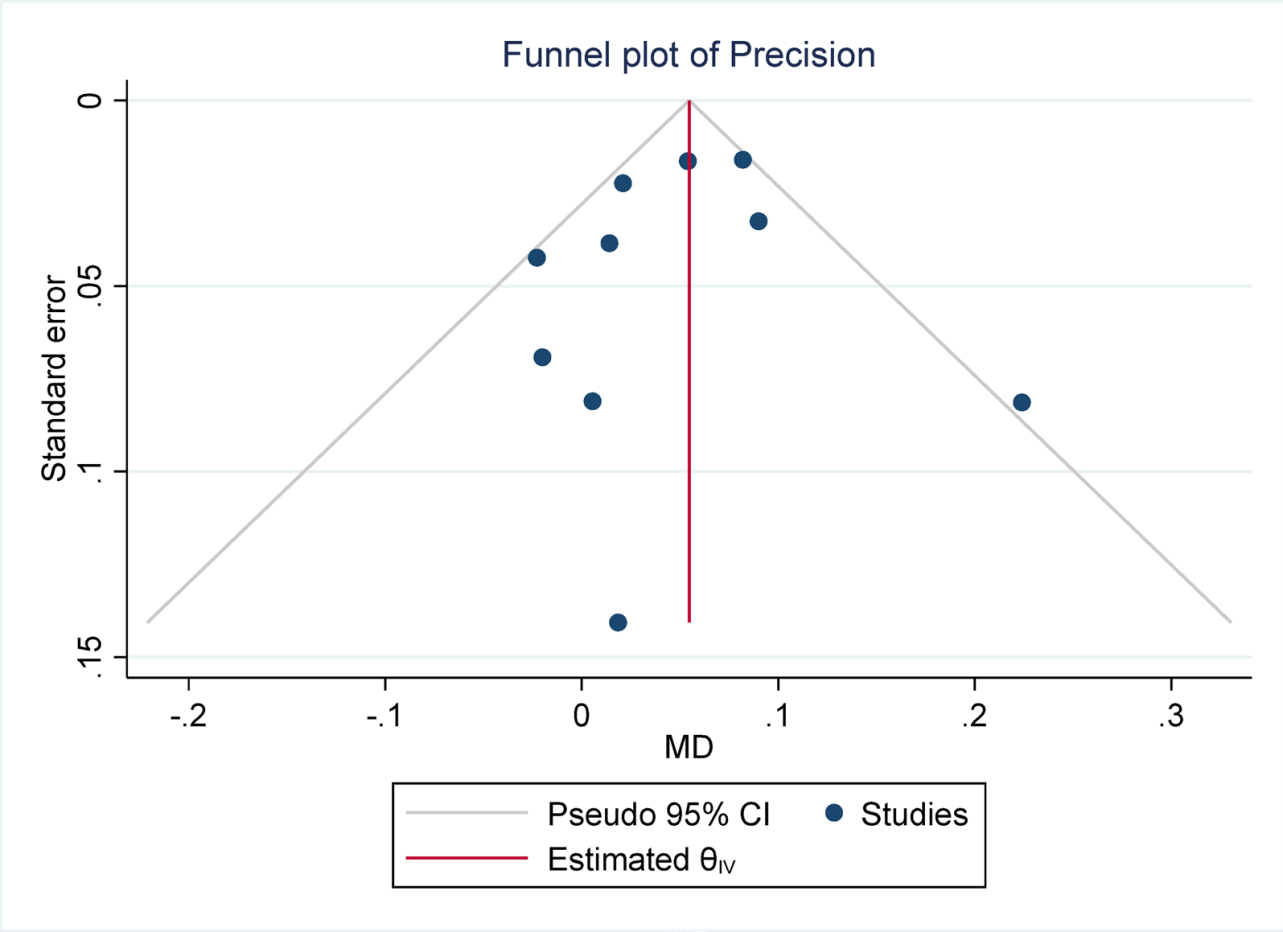
Funnel plot for precision. The Egger test indicated no significant publication bias (*p* = 0.732)

**Fig. 13:**
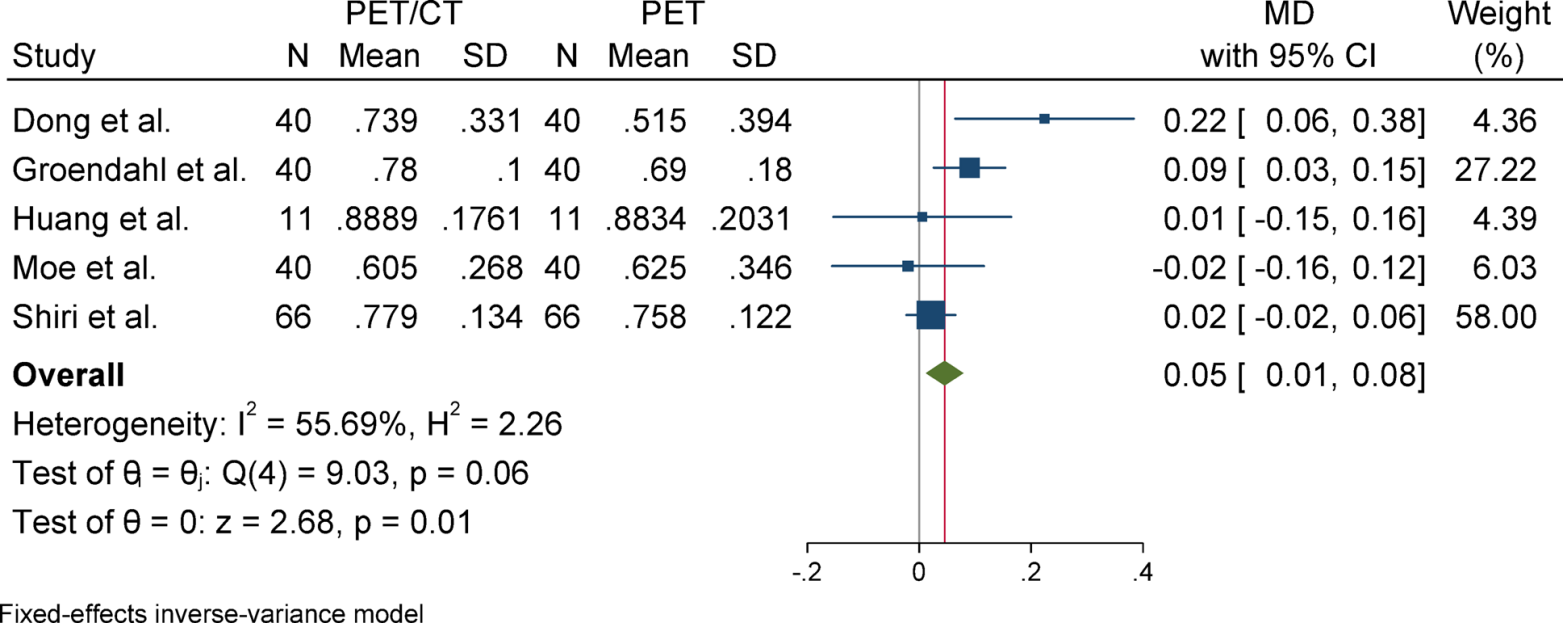
Forest plot for precision after removing SD-imputed studies. The overall mean difference remained relatively stable compared to the initial analysis using imputed SDs, indicating the accuracy and robustness of the SD estimation method

**Fig. 14:**
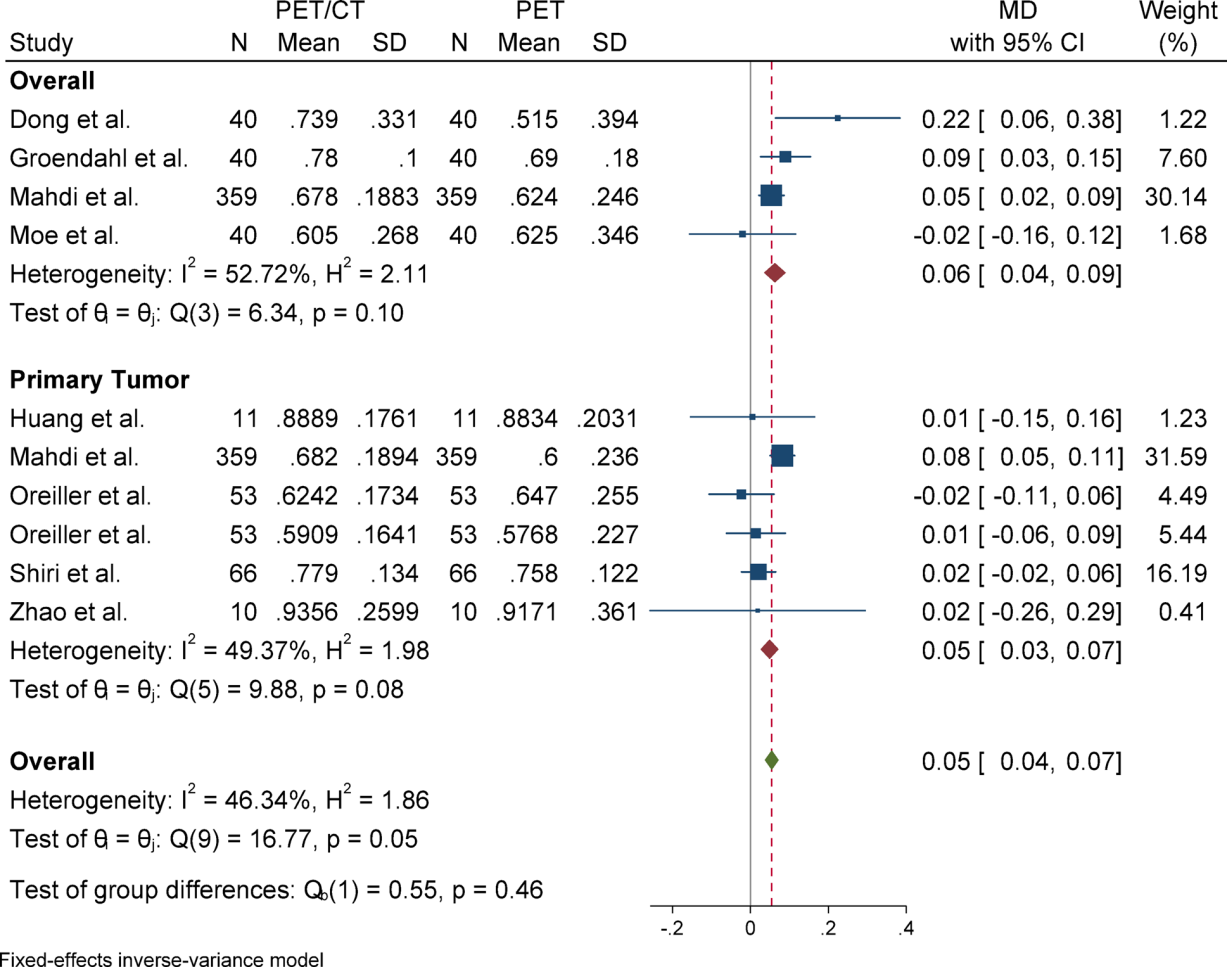
Subgroup analyses of precision in terms of overall and primary tumor segmentation

**Fig. 15:**
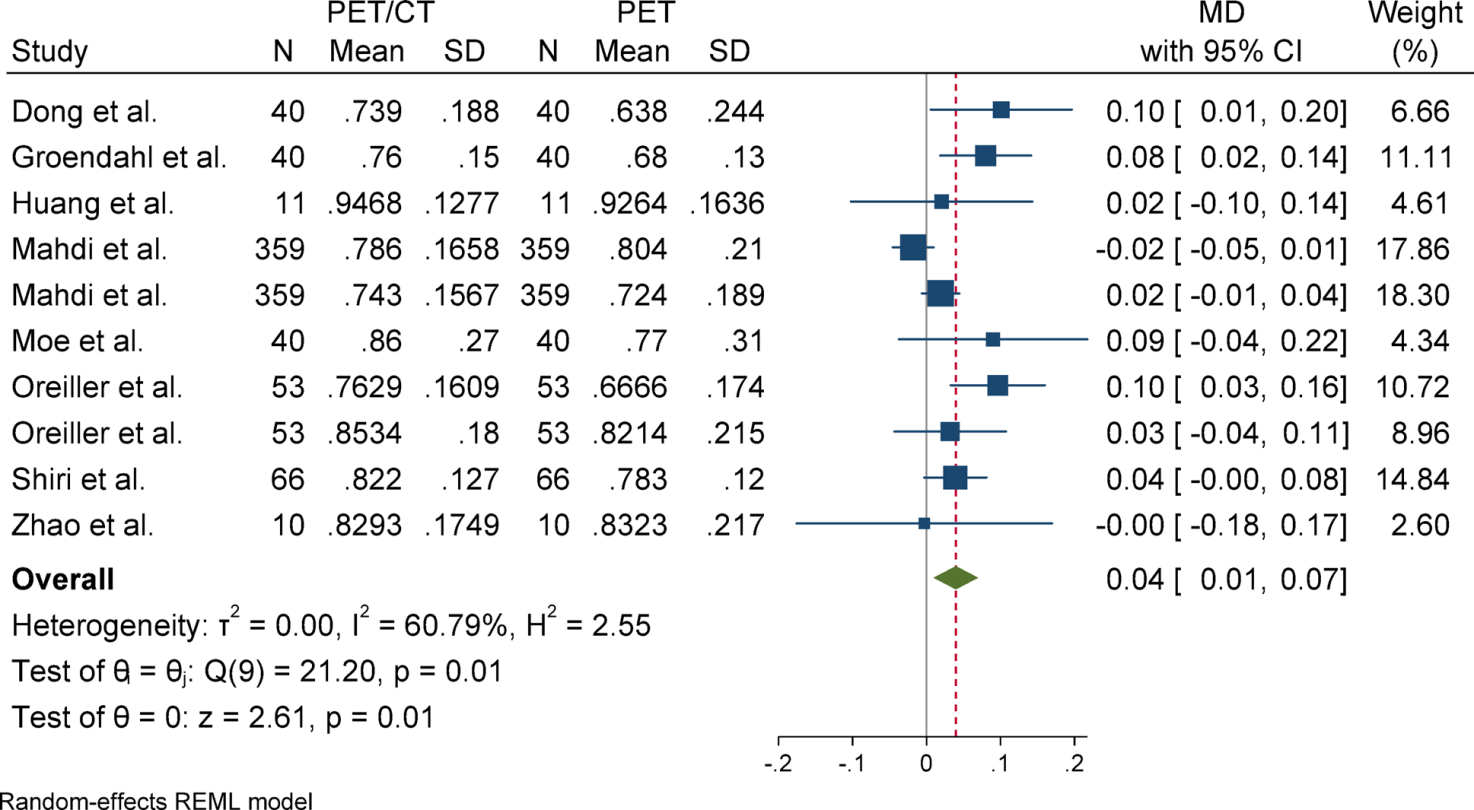
Forest plot for sensitivity; Forest plot shows the pooled mean difference (MD) of sensitivity between PET/CT and PET across 14 studies, favoring PET/CT (MD = 0.04, 95% CI 0.01 to 0.07) with low heterogeneity (I^2^ = 60.79%)

**Fig. 16:**
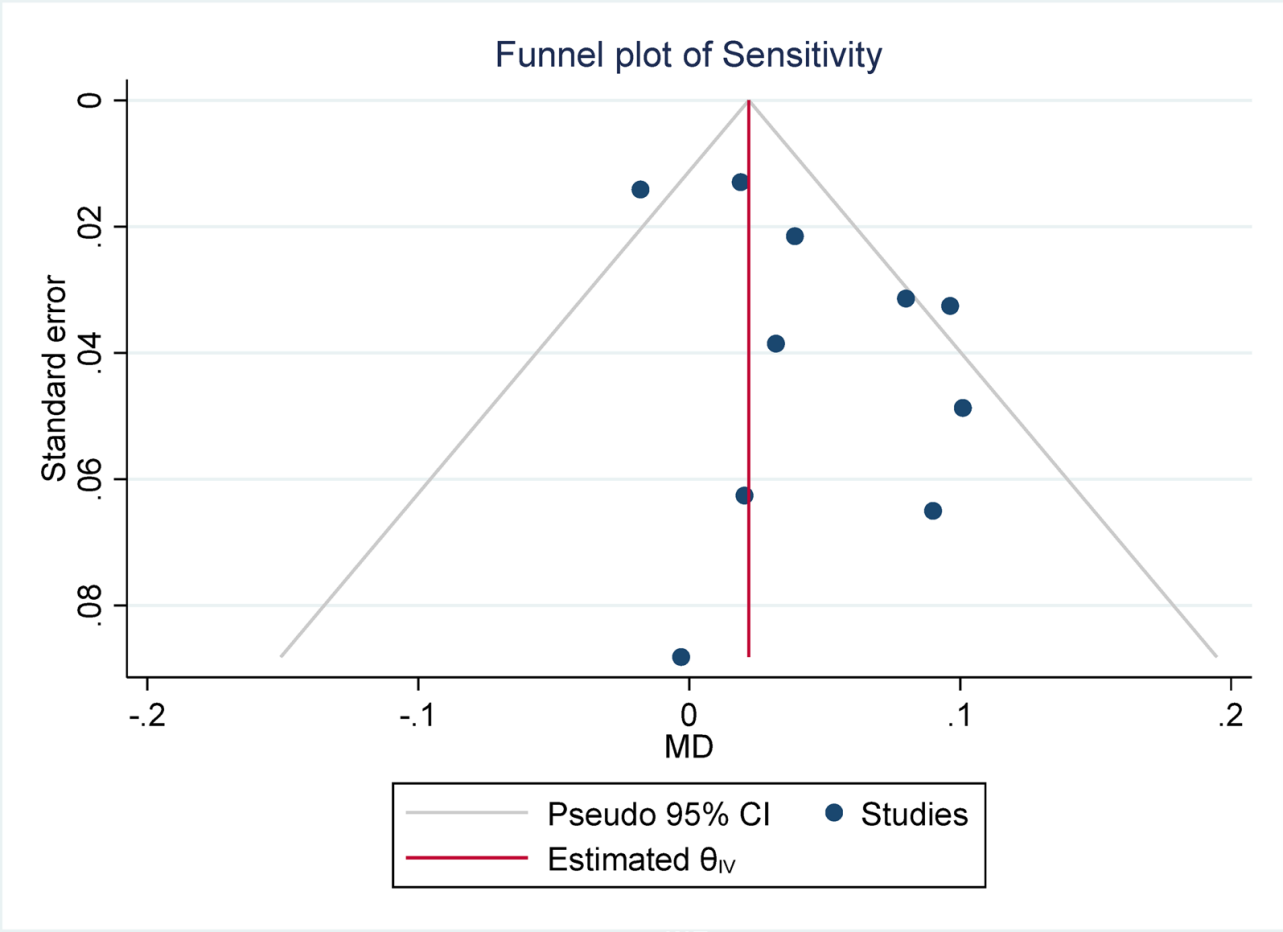
Funnel plot for precision. The Egger test indicated no significant publication bias (*p* = 0.148)

**Fig. 17:**
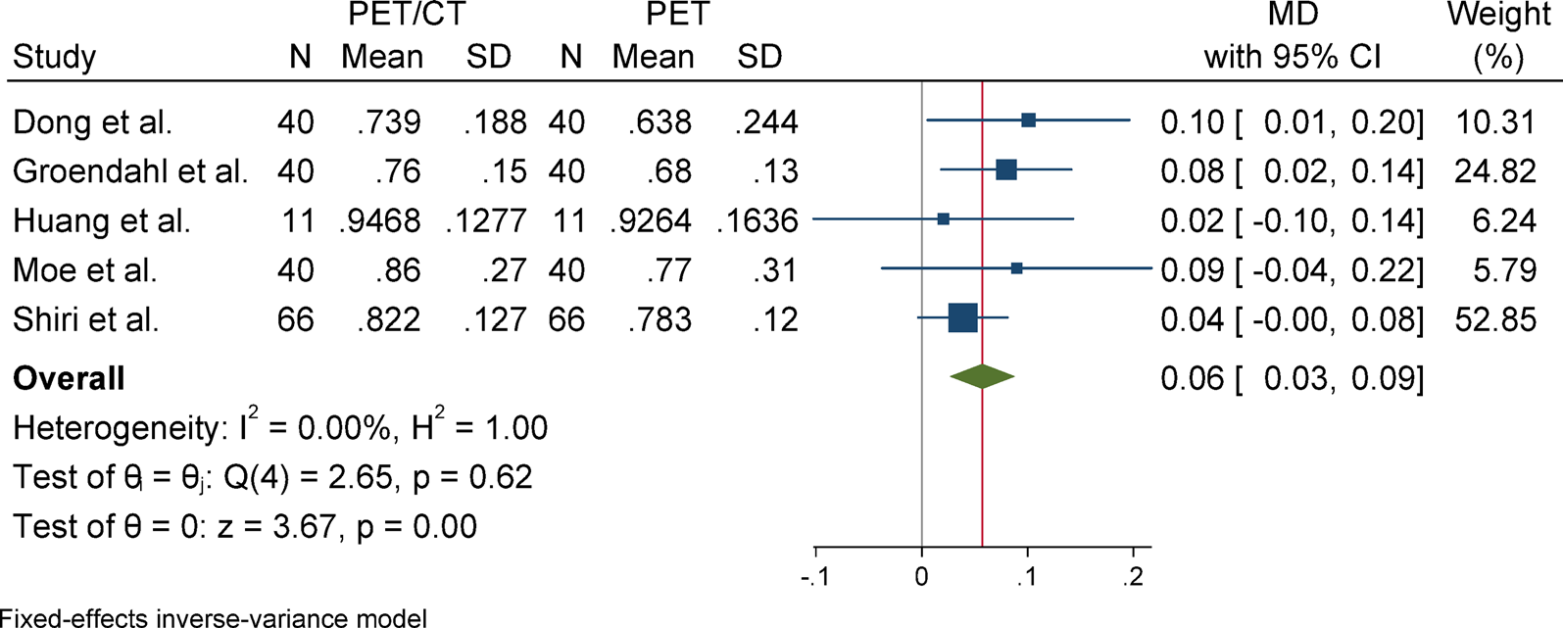
Forest plot for sensitivity after removing SD-imputed studies. The overall mean difference remained relatively stable compared to the initial analysis using imputed SDs, indicating the accuracy and robustness of the SD estimation method

**Fig. 18:**
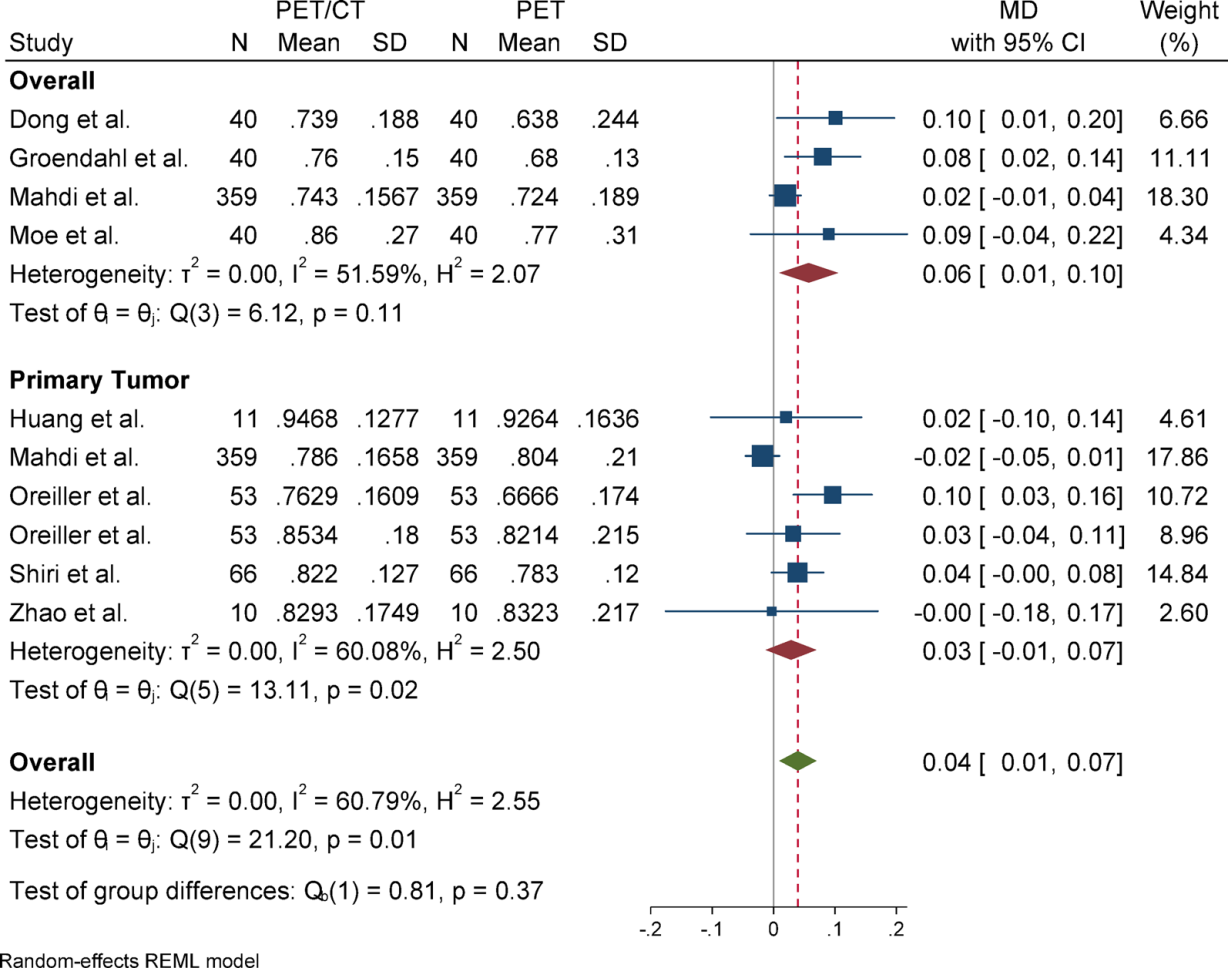
Subgroup analyses of sensitivity in terms of overall and primary tumor segmentation

**Fig. 19:**
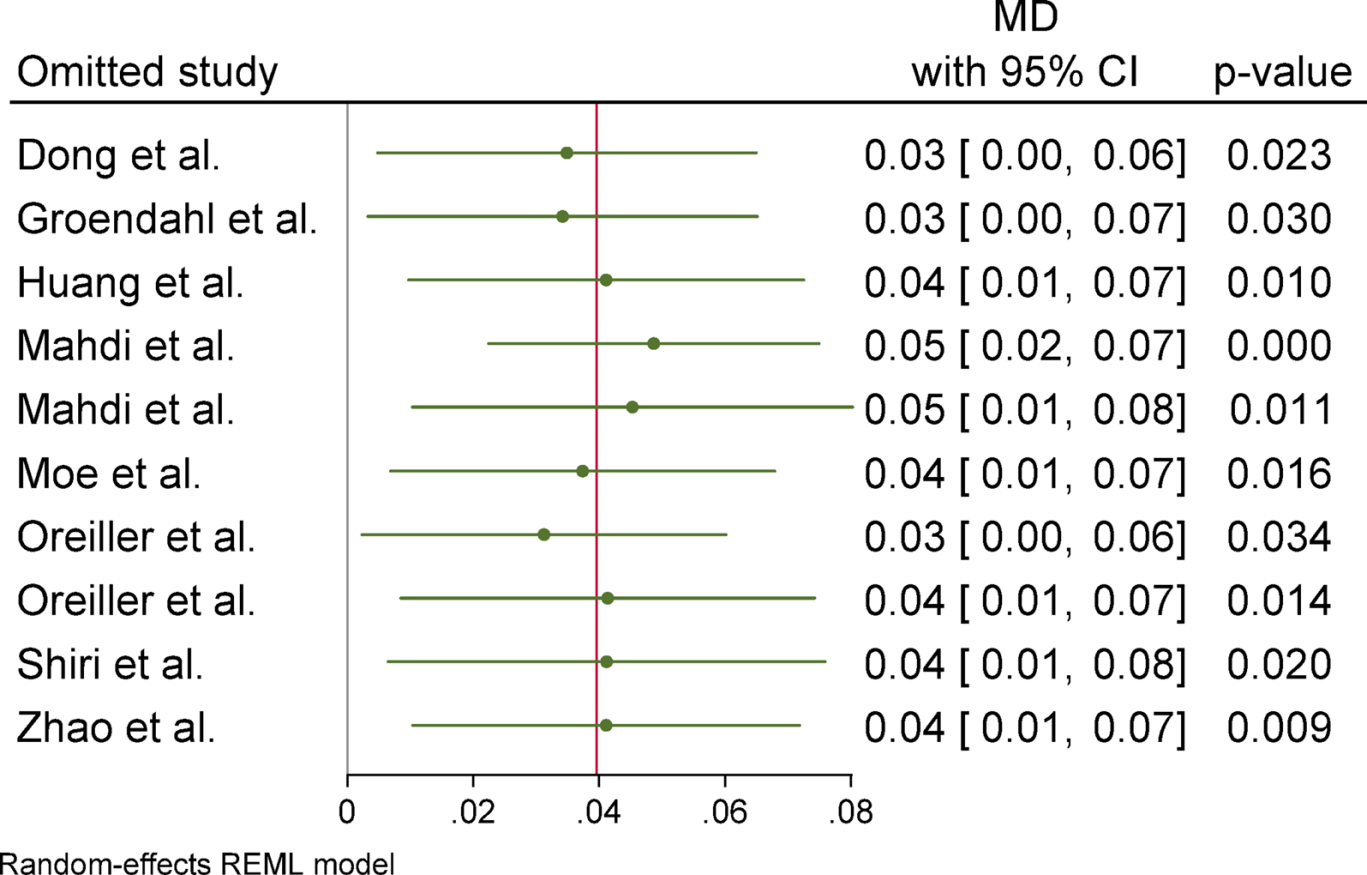
One-leave-out analysis for sensitivity

Subgroup analyses were conducted based on segmentation target (overall vs. primary tumor) to explore potential sources of heterogeneity. For DSC and Precision, both subgroups yielded statistically significant results with low heterogeneity. In contrast, the HD metric showed considerable heterogeneity in the overall group but not in the primary tumor group, although neither subgroup reached statistical significance. For Sensitivity, the overall group showed a significant effect, while the primary tumor group did not, despite significant heterogeneity in the latter. These patterns highlight the influence of segmentation targets on outcome variability and underscore the need for methodological standardization across studies (Figs. 2, 3, 4, 5, 6, 7, 8, 9, 10, 11, 12, 13, 14, 15, 16, 17, 18, 19).

## Discussion

This meta-analysis indicates that AI models used in the segmentation of head and neck tumors demonstrate remarkable improvement in performance when used with PET/CT, as opposed to only using the PET modality. Concerning quantifiable data, all metrics were improved by PET/CT, including an increment of approximately 0.05 in the Dice Similarity Coefficient (DSC), along with a 0.05 increase in precision and 0.04 in sensitivity, which denotes more adequate tumor identification. Furthermore, PET/CT demonstrated an improvement in the accuracy of boundary delineation errors, as shown by a decrease of approximately 3 mm in Hausdorff Distance (HD95). These changes were stable, as low heterogeneity was demonstrated for the primary metrics, implying that the superiority of PET/CT is likely consistent.

The pooled Dice coefficients reported in our meta-analysis reflect a high degree of spatial agreement between automated and manual segmentations. In radiotherapy planning, Dice scores above 0.80 are generally considered clinically acceptable, as they suggest sufficient contour accuracy to support target delineation. Similarly, the observed sensitivity values above 90% in several models indicate strong tumor coverage, minimizing the risk of under-segmentation and potential geographical miss during treatment [40]. In clinical practice, automated segmentation models with acceptable accuracy (e.g., Dice coefficient ≥ 0.80) can support radiotherapy planning by reducing manual workload and interobserver variability. Several studies have demonstrated that auto-segmented contours serve as reliable initial contours for expert revision, significantly improving clinical efficiency. Given the observed accuracy levels in our analysis, these models hold promise for integration into clinical workflows, particularly in resource-limited settings where expert availability is constrained [41].

The findings from this meta-analysis support the previously included primary studies in our review, which show that PET/CT has a marked advantage over PET alone in AI-based segmentation of head and neck tumors. Dong [24] reported a DSC of 0.739 with 3D diffusion-based models on PET/CT, which is reasonably close to our pooled estimate. Groendahl [35] observed that dual-modality CNN models performed better than the thresholding methods using PET only, with DSC increasing from 0.68 to 0.74. Guo [36] further proved this trend using dense multi-modality frameworks. More recent work by Mahdi [32] with Weighted Fusion Transformer and Huang [22] with ISA-Net have shown surpassing 0.80 DSC, which points towards an increased efficacy of multimodal fusion. Moe [38] and Oreiller [26] showed that extracting models based on combined PET/CT for HECKTOR datasets results in fewer boundary errors and improved coverage fractions. Shiri [28] reported lower false negative rates and greater robustness via hybrid level fusion. Zhao [39] conversely showed that PET-only FCNs do not reach comparable accuracy and sensitivity in segmentation. Also, in the second study by Zhao [20] the MMCA-Net cross-attention-based transformer network was introduced, which outperformed all ten comparative algorithms with a mean DSC of 0.815. These results from different architectures, modalities, and sites of tumors support the statement that AI-based segmentation of PET/CT modality offers enhanced performance metrics with general applicability to multiple clinical scenarios.

The superiority of PET/CT in AI-based segmentation of head and neck tumors is likely due to the integration of anatomical structures and metabolism, which enhances boundary refinement and decreases false positive segmentations [42, 43]. PET provides valuable functional information, particularly in the intricate contours of some anatomical regions, but it is usually not accompanied by clear spatial resolution. CT improves structural understanding and is more useful in morphologic segmentation when used with deep learning models that integrate with PET [44, 45]. In addition to morphological and textural characterization, Vision Transformers (VIT)’s classification of low-FDG lesions demonstrates the effectiveness of using two imaging modalities and emphasizes the advanced radiomic features of PET/CT [46, 47].

CT’s role in PET/CT imaging also assists in prognostic modeling and extends the applicability of radiomics-based AI solutions, especially in feature constancy within spatially complex tumors [48, 49]. The integration of CT with PET enhances the stability of radiomic signatures and supports patient stratification based on tumor geometry and metabolic profile [50]. Furthermore, AI-driven PET/CT contouring reduces inter-observer variability and enables more consistent identification of organs at risk during radiotherapy planning [51]. In general, multimodal PET/CT improves the accuracy and trustworthiness of tumor delineation AI models, which facilitates radiotherapy treatment planning [52]. Finally, delays in treatment initiation—often due to inadequate imaging precision or planning inefficiencies—have been shown to significantly increase cancer-specific mortality, emphasizing the value of early, AI-assisted PET/CT-based planning to support more time-sensitive and effective oncologic care [53, 54].

Several sources of heterogeneity among the included studies warrant consideration. First, tumor site may influence segmentation difficulty, as regions such as the nasopharynx or larynx present greater anatomical complexity than, for instance, the oral cavity. Second, different deep learning architectures were used—primarily CNN-based and Transformer-based models—which may explain variations in segmentation performance, as Transformer models often yield better global context understanding but require larger datasets. Lastly, dataset characteristics, including imaging modality (PET vs. PET/CT), annotation protocols, and dataset size, varied substantially across studies and may have influenced model generalizability. These factors collectively contribute to the observed heterogeneity and should be considered when interpreting pooled performance metrics.

## Limitations

This systematic review and meta-analysis was based on 11 studies only, decreasing the statistical power and increasing the effect of possible outliers. One major limitation of our meta-analysis is the pooling of results across tumors from anatomically diverse regions (e.g., nasopharynx, oropharynx, parotid gland). These sites differ in imaging characteristics and segmentation difficulty, which may introduce heterogeneity into performance metrics. Although subgroup analysis was not feasible due to data limitations, this should be considered when interpreting the pooled results. Although random-effects models were used and Egger’s tests did not indicate small-study effects, the limited number of included studies diminishes the ability to perform comprehensive subgroup analyses, especially regarding the variations of AI algorithms used. Furthermore, the imputation of missing standard deviations in some cases added more uncertainty to the pooled estimates. Differences in reported outcomes, including whether segmentation focused on primary tumors or total tumor burden, also restrict comparability across the studies. Nevertheless, these findings are crucial from a clinical perspective. AI-assisted segmentation of gross tumor volume (GTV) using PET/CT and AI is superior to PET alone, which is particularly important for radiotherapy planning in dose-escalation and early adaptive treatment scenarios. AI also diminishes inter-observer variability, leading to streamlined workflows at varying institutions, thus enhancing standardization. The success of endeavors such as the HECKTOR challenge demonstrates the utility of publicly available PET/CT databases in training and validation. Subsequently, future research should focus more on federated learning that allows the sharing of sensitive information safely, and focus on predictive models by including uncertainty quantification.

## Conclusions

This systematic review and meta-analysis demonstrates with great certainty that artificial intelligence models that employ PET/CT imaging perform significantly better regarding head and neck tumor segmentation than models that only use PET imaging. In particular, approaches that used PET/CT demonstrated better performance in all the primary metrics, including Dice Similarity Coefficient, sensitivity, precision, and accuracy of boundary delineation, which explains the advantage of integrating both metabolic and structural information on head and neck anatomy for better tumor localization. These gains were achieved with different datasets and AI architectures, suggesting applicability in practice and remote settings. Although there are some issues related to the heterogeneity of studies, sample size, and differences in methods used, the overall conclusions encourage the adoption of AI segmentation approaches using PET/CT in clinical practice. With the evolving research, the focus should be on the need for documentation of reporting and measuring uncertainty and the creation of multi-institutional repositories to foster ease of access, consistency, and practical value of research output. Further research should be conducted on using federated learning and segmentation-plus models that not only define the regions of interest but also shift AI use towards the aims of personalized outcome forecasting in precision oncology.

## Supplementary Information

Below is the link to the electronic supplementary material.

Supplementary Material 1

## Data Availability

The datasets used and/or analysed during the current study are available from the corresponding author on reasonable request.

## Abbreviations

AI: Artificial intelligence
PET: Positron emission tomography
CT: Computed tomography
PET/CT: Positron emission tomography/computed tomography
HNC: Head and neck cancer
CNN: Convolutional neural network
GTV: Gross tumor volume
GTVt: Gross tumor volume of the primary tumor
GTVn: Gross tumor volume of the lymph nodes
DSC: Dice similarity coefficient
HD95: 95th percentile of Hausdorff distance
PPV: Positive predictive value
TPR: True positive rate
SD: Standard deviation
SE: Standard error
MD: Mean difference
CI: Confidence interval
PI: Prediction interval
I^2^: I-squared statistic
τ^2^: Between-study variance
QUADAS-C: Quality assessment of diagnostic accuracy studies-comparative
CLAIM: Checklist for artificial intelligence in medical imaging
PRISMA-DTA: Preferred reporting items for systematic reviews and meta-analyses of diagnostic test accuracy studies
REML: Restricted maximum likelihood
DL: DerSimonian–Laird
WLS: Weighted least squares
FCN: Fully convolutional network
STS: Soft tissue sarcoma
ASSD: Average symmetric surface distance
MSD: Mean surface distance
VOE: Volume overlap error
RVD: Relative volume difference
HECKTOR: Head and neck tumor segmentation and outcome prediction in PET/CT images challenge
MICCAI: Medical image computing and computer-assisted intervention
