# Supplementary material for: Application of artificial intelligence in head and neck tumor segmentation: a comparative systematic review and meta-analysis between PET and PET/CT modalities
Source: BMC Cancer. 2025 Oct 27;25:1656. doi: 10.1186/s12885-025-14881-8 (PMC12560352; doi:10.1186/s12885-025-14881-8)
Supplement: Supplementary file 1 — Supplementary Material 1 [file 12885_2025_14881_MOESM1_ESM.docx]

**Supplementary:**

| **Database** | **Keywords** | **Results** |
| --- | --- | --- |
| **Scopus** | TITLE-ABS-KEY(("Artificial Intelligence" OR "Machine Learning" OR "Deep Learning" OR "Neural Network" OR "AI") AND ("head" OR "Neck" OR "oral cavity" OR "pharynx" OR "larynx" OR "nasal cavity" OR "sinus" OR "salivary gland" OR "oropharyngeal" OR "nasopharyngeal" OR "oropharynx" OR "nasopharynx" OR "hypopharynx" OR "head and neck" OR "HNC" OR "head & neck") AND ("cancer" OR "tumor" OR "malignancy" OR "neoplasm" OR "carcinoma") AND ("Positron-Emission Tomography" OR "Tomography, Emission-Computed" OR "Fluorodeoxyglucose F18" OR "PET" OR "Positron Emission Tomography" OR "FDG-PET" OR "18F-FDG PET" OR "PET/CT")) | 481 |
| **Embase** | ('Artificial Intelligence' OR 'Machine Learning' OR 'Deep Learning' OR 'Neural Network' OR 'AI')  AND ('head' OR 'Neck' OR 'oral cavity' OR 'pharynx' OR 'larynx' OR 'nasal cavity' OR 'sinus' OR 'salivary gland' OR 'oropharyngeal' OR 'nasopharyngeal' OR 'oropharynx' OR 'nasopharynx' OR 'hypopharynx' OR 'head and neck' OR 'HNC' OR 'head & neck') AND ('cancer' OR 'tumor' OR 'malignancy' OR 'neoplasm' OR 'carcinoma') AND ('Positron-Emission Tomography' OR 'Tomography, Emission-Computed' OR 'Fluorodeoxyglucose F18' OR 'PET' OR 'Positron Emission Tomography' OR 'FDG-PET' OR '18F-FDG PET' OR 'PET/CT') | 702 |
| **PubMed** | (("Artificial Intelligence"[MeSH Terms] OR "Artificial Intelligence"[All Fields] OR "Machine Learning"[All Fields] OR "Deep Learning"[All Fields] OR "Neural Network"[All Fields] OR "AI"[All Fields]) AND ("Head and Neck Neoplasms"[MeSH Terms] OR "Head and Neck"[All Fields] OR "HNC"[All Fields] OR "head & neck"[All Fields] OR "oral cavity"[All Fields] OR "pharynx"[All Fields] OR "larynx"[All Fields] OR "nasal cavity"[All Fields] OR "sinus"[All Fields] OR "salivary gland"[All Fields] OR "oropharyngeal"[All Fields] OR "nasopharyngeal"[All Fields] OR "oropharynx"[All Fields] OR "nasopharynx"[All Fields] OR "hypopharynx"[All Fields]) AND ("Neoplasms"[MeSH Terms] OR "cancer"[All Fields] OR "tumor"[All Fields] OR "malignancy"[All Fields] OR "neoplasm"[All Fields] OR "carcinoma"[All Fields]) AND ("Positron-Emission Tomography"[MeSH Terms] OR "Tomography, Emission-Computed"[MeSH Terms] OR "Fluorodeoxyglucose F18"[MeSH Terms] OR "Positron Emission Tomography"[All Fields] OR "PET"[All Fields] OR "FDG-PET"[All Fields] OR "18F-FDG PET"[All Fields] OR "PET/CT"[All Fields])) | 296 |
| **Cochrane** | ("Artificial Intelligence" OR "Machine Learning" OR "Deep Learning" OR "Neural Network" OR "AI") AND ("head" OR "Neck" OR "oral cavity" OR "pharynx" OR "larynx" OR "nasal cavity" OR "sinus" OR "salivary gland" OR "oropharyngeal" OR "nasopharyngeal" OR "oropharynx" OR "nasopharynx" OR "hypopharynx" OR "head and neck" OR "HNC" OR "head & neck") AND ("cancer" OR "tumor" OR "malignancy" OR "neoplasm" OR "carcinoma") AND ("Positron-Emission Tomography" OR "Tomography, Emission-Computed" OR "Fluorodeoxyglucose F18" OR "PET" OR "Positron Emission Tomography" OR "FDG-PET" OR "18F-FDG PET" OR "PET/CT") | 14 |
| **Web of Science** | TS=("Artificial Intelligence" OR "Machine Learning" OR "Deep Learning" OR "Neural Network" OR "AI") AND TS=("head" OR "Neck" OR "oral cavity" OR "pharynx" OR "larynx" OR "nasal cavity" OR "sinus" OR "salivary gland" OR "oropharyngeal" OR "nasopharyngeal" OR "oropharynx" OR "nasopharynx" OR "hypopharynx" OR "head and neck" OR "HNC" OR "head & neck") AND TS=("cancer" OR "tumor" OR "malignancy" OR "neoplasm" OR "carcinoma") AND TS=("Positron-Emission Tomography" OR "Tomography, Emission-Computed" OR "Fluorodeoxyglucose F18" OR "PET" OR "Positron Emission Tomography" OR "FDG-PET" OR "18F-FDG PET" OR "PET/CT") | 289 |
| **Google Scholar** | allintitle:("Artificial Intelligence" OR "Machine Learning" OR "Deep Learning" OR "Neural Network" OR "AI")  ("head" OR "Neck" OR "oral cavity" OR "pharynx" OR "larynx" OR "nasal cavity" OR "sinus" OR "salivary gland" OR "oropharyngeal" OR "nasopharyngeal" OR "oropharynx" OR "nasopharynx" OR "hypopharynx" OR "head and neck" OR "HNC" OR "head & neck")  ("cancer" OR "tumor" OR "malignancy" OR "neoplasm" OR "carcinoma")  ("Positron-Emission Tomography" OR "Tomography, Emission-Computed" OR "Fluorodeoxyglucose F18" OR "PET" OR "Positron Emission Tomography" OR "FDG-PET" OR "18F-FDG PET" OR "PET/CT") | 200  (First 200 results) |

Supplementary 1-Specific search query for databases

| Ren et al., 2021 | Comparing different CT, PET and MRI multi-modality image combinations for deep learning-based head and neck tumor segmentation | Because it lacks a standalone CT or standalone PET arm (and thus does not fulfill the “compare at least two of PET/CT, CT, PET” requirement in a quantitative, head-to-head manner), this paper should be Excluded. |
| --- | --- | --- |
| Ren et al., 2020 | Deep learning delineation of GTV for head and neck cancer with multi-modality imaging | Because this study never evaluates CT alone or PET alone, it does not fulfill the requirement of comparing those modalities in isolation. |
| Wang et al., 2020 | Comparison of deep learning networks for fully automated head and neck tumor delineation on multi-centric PET/CT images | Because it lacks a comparison of at least two of the specified modalities (PET/CT, CT, PET) in isolation or against each other, it does not meet the inclusion criteria. |
| Cho et al., 2024 | Multi-modal co-learning with attention mechanism for head and neck tumor segmentation on 18FDG PET-CT | Because it does not compare at least two of the specified imaging modalities in isolation or against one another, it fails the modality-comparison requirement and should be Excluded. |
| Rainio et al., 2024 | One-click annotation to improve segmentation by a convolutional neural network for PET images of head and neck cancer patients | Because the study does not include or compare at least two of the specified imaging modalities (CT, PET, PET/CT) in a quantitative way, it does not meet the modality-comparison requirement and should be Excluded. |
| Zhao et al., 2024 | Multi-modal segmentation with missing image data for automatic delineation of gross tumor volumes in head and neck cancers | it compares PET/CT with CT |
| Li et al., 2024 | Efficient model-informed co-segmentation of tumors on PET/CT driven by clustering and classification information | Because it lacks a direct, numerical comparison of the specified imaging modalities in isolation, it fails the modality‐comparison criterion and should be Excluded. |
| Huynh et al., 2024 | Deep learning with uncertainty estimation for automatic tumor segmentation in PET/CT of head and neck cancers: impact of model complexity, image processing and augmentation | Because it lacks a direct, quantitative comparison of the specified imaging modalities in isolation, it fails the modality-comparison criterion and should be Excluded. |
| Ren et al., 2024 | Enhancing the reliability of deep learning-based head and neck tumour segmentation using uncertainty estimation with multi-modal images | Because it does not quantitatively compare two or more of the specified imaging modalities in isolation, it fails the modality-comparison criterion and should be Excluded. |
| De Biase et al., 2024 | Probability maps for deep learning-based head and neck tumor segmentation: Graphical User Interface design and test | Because it does not meet the requirement of quantitatively comparing at least two of the specified imaging modalities, this article should be Excluded. |
| Fukushima et al., 2024 | Evaluation of bone marrow invasion on the machine learning of 18F-FDG PET texture analysis in lower gingival squamous cell carcinoma | Because it fails to compare at least two of the specified imaging modalities in a quantitative manner, it does not meet the inclusion criteria. |
| Lechien et al., 2024 | Accuracy of ChatGPT in head and neck oncological board decisions: preliminary findings | Because it does not involve comparative imaging analysis between the specified modalities using quantitative metrics, it fails the modality-comparison criterion and should be Excluded. |
| van Staalduinen et al., 2023 | Improved Cervical Lymph Node Characterization among Patients with Head and Neck Squamous Cell Carcinoma Using MR Texture Analysis Compared to Traditional FDG-PET/MR Features Alone | Because it lacks a central AI/ML methodology and does not compare at least two of PET/CT, CT, and PET with objective metrics, it does not meet the inclusion criteria. |
| Santer et al., 2024 | The Neck-Persistency-Net: a three-dimensional, convolution, deep neural network aids in distinguishing vital from non-vital persistent cervical lymph nodes in advanced head and neck squamous cell carcinoma after primary concurrent radiochemotherapy | Fails to quantitatively compare at least two imaging modalities (e.g., PET vs. CT or PET/CT vs. CT) using objective metrics. |
| Fan et al., 2024 | A medical image classification method based on self-regularized adversarial learning | Because it fails to compare at least two specified imaging modalities in a quantitative manner, it does not meet the inclusion criteria. |
| Kovacs et al., 2024 | Clinical Evaluation of Deep Learning for Tumor Delineation on 18F-FDG PET/CT of Head and Neck Cancer | Because it lacks a comparison of at least two of the specified imaging modalities, it does not meet the inclusion criteria and should be Excluded. |
| Leung et al., 2024 | Deep Semisupervised Transfer Learning for Fully Automated Whole-Body Tumor Quantification and Prognosis of Cancer on PET/CT | The study includes multiple cancer types (lung, melanoma, lymphoma, breast, prostate, and head and neck). While head and neck cancer is mentioned, it is not the sole focus of the research. |
| Qasem et al., 2025 | Automated tumor localization and segmentation through hybrid neural network in head and neck cancer | Despite its focus on head and neck cancer and advanced AI methodology, the article is excluded because it does not meet the requirement for direct, numerical comparison of imaging modalities (e.g., segmentation accuracy of PET vs. CT). The comparisons are limited to algorithm performance, not modality performance. |
| Liedes et al., 2023 | Automatic Segmentation of Head and Neck Cancer from PET-MRI Data Using Deep Learning | Though the article focuses on head and neck cancer and employs AI for segmentation, it is excluded because it does not quantitatively compare PET/CT, CT, or PET as standalone modalities. The comparison between PET and PET-MRI falls outside the scope of the inclusion criteria. |
| Shiri et al., 2023 | Multi-institutional PET/CT image segmentation using federated deep transformer learning | While the article focuses on head and neck cancer and employs advanced AI (federated transformer learning), it does not meet the requirement for direct, numerical comparison of imaging modalities (e.g., segmentation accuracy of PET vs. CT). The comparisons are limited to learning frameworks, not modality performance. Thus, it is excluded. |
| Wu et al., 2023 | Locally advanced nasopharyngeal carcinoma gross tumor volume auto-segmentation on planning CT with multi-modality image learning | Although the article focuses on head and neck cancer and employs AI for segmentation, it is excluded because it does not meet the requirement for direct, numerical comparison of PET/CT, CT, or PET as standalone modalities. The study evaluates a multi-modality fusion approach rather than comparing the diagnostic or segmentation performance of individual modalities. |
| Nikulin et al., 2023 | A convolutional neural network with self-attention for fully automated metabolic tumor volume delineation of head and neck cancer in [18 F]FDG PET/CT | Although the article focuses on head and neck cancer and employs advanced AI for tumor segmentation, it is excluded because it does not meet the requirement for direct, numerical comparison of imaging modalities (e.g., PET vs. CT or PET/CT vs. CT). The study evaluates model performance on PET/CT as a fused input rather than comparing modalities. |
| Hellström et al., 2023 | Classification of head and neck cancer from PET images using convolutional neural networks | While the article addresses head and neck cancer and uses AI for classification, it is excluded because it does not meet the requirement for direct, numerical comparison of imaging modalities (e.g., PET vs. CT or PET/CT vs. PET). The analysis is confined to PET-based model performance, with no evaluation of other modalities. |
| Salmanpour et al., 2023 | Prediction of TNM Stage in Head and NeckCancer Using Tensor Deep vs. RadiomicsFeatures | Because it fails to address the core task (diagnosis/segmentation) and doesn’t include a quantitative modality comparison on the same outcome, it should be Excluded. |
| Samanta et al., 2022 | Federated Learning on 18F-FDG PET/CT Uptake Classification in Lung Cancer, Lymphoma and Head and Neck Cancer | Conference Paper |
| Shiri et al., 2022 | Multi-Institutional PET/CT Image Segmentation Using a Decentralized Federated Deep Transformer Learning Algorithm | Although the article focuses on head and neck cancer and employs advanced AI (federated transformer learning), it is excluded because it does not meet the requirement for direct, numerical comparison of imaging modalities (e.g., PET vs. CT or PET/CT vs. CT). The study evaluates algorithm performance rather than modality performance. |
| Cho et al., 2022 | Improved performance of head and neck tumor segmentation in FDG PET/CT using an integrated attention network | Conference Paper/ Inadequate data |
| Xu et al., 2023 | Radiomics prognostic analysis of PET/CT images in a multicenter head and neck cancer cohort: investigating ComBat strategies, sub-volume characterization, and automatic segmentation | While the article addresses head and neck cancer and employs AI/ML for prognosis and segmentation, it is excluded because it does not meet the requirement for direct, numerical comparison of imaging modalities (e.g., PET vs. CT or PET/CT vs. CT). The analysis is confined to methodological refinements within PET/CT, not modality performance. |
| Ren et al., 2023 | Single-click user input reduces false detection in deep learning head and neck tumor segmentation | Although the article addresses head and neck cancer and employs AI for segmentation, it is excluded because it does not meet the requirement for direct, numerical comparison of imaging modalities (e.g., PET vs. CT or PET/CT vs. CT). The analysis focuses on user interaction improvements and multi-modal input fusion, not modality performance. |
| Bollen et al., 2023 | Clinical benefits of multi-modality gross tumor volume auto-delineation in head and neck cancer | comparing with MRI |
| Salahuddin et al., 2023 | From Head and Neck Tumour and Lymph Node Segmentation to Survival Prediction on PET/CT: An End-to-End Framework Featuring Uncertainty, Fairness, and Multi-Region Multi-Modal Radiomics | While the article addresses head and neck cancer and employs advanced AI/ML techniques, it is excluded because it does not meet the requirement for direct, numerical comparison of imaging modalities (e.g., PET vs. CT or PET/CT vs. CT). The analysis focuses on multi-modal fusion and survival prediction, not modality-specific performance. |
| Zhou et al., 2023 | MRLA-Net: A tumor segmentation network embedded with a multiple receptive-field lesion attention module in PET-CT images | Does not exclusively focus on head and neck cancer (violating the first inclusion criterion). Lacks a direct, numerical comparison of PET, CT, or PET/CT modalities. The study evaluates network architecture improvements, not modality performance. |
| Woo et al., 2023 | Development and Testing of a Machine Learning Model Using18F-Fluorodeoxyglucose PET/CT-Derived Metabolic Parameters to Classify Human Papillomavirus Status in Oropharyngeal Squamous Carcinoma | While the article focuses on head and neck cancer and employs AI/ML, it does not meet the requirement for direct, numerical comparison of imaging modalities (e.g., PET vs. CT or PET/CT vs. CT). The analysis centers on feature type comparisons (PET parameters vs. clinical data) rather than modality performance. Thus, it is excluded. |
| Wei et al., 2023 | Towards interactive deep-learning for tumour segmentation in head and neck cancer radiotherapy | While the article addresses head and neck cancer and employs AI/ML for segmentation, it is excluded because it does not meet the requirement for direct, numerical comparison of imaging modalities (e.g., PET vs. CT or PET/CT vs. CT). The analysis focuses on interactive learning improvements, not modality-specific segmentation performance. |
| Henson et al., 2023 | Diagnostic challenges and prognostic implications of extranodal extension in head and neck cancer: a state of the art review and gap analysis | While the article is relevant to head and neck cancer, it is excluded because it is a review paper and does not meet the criteria for AI/ML centrality or quantitative imaging modality comparisons. |
| Dohopolski et al., 2022 | Using Radiomics to Improve the Diagnostic Accuracy of Indeterminate Residual Primary Disease on Restaging PET/CT Imaging Following Radiation Therapy for Head and Neck Cancers | Conference Paper/ Inadequate data |
| Shiri et al., 2022 | Decentralized Distributed Multi-institutional PET Image Segmentation Using a Federated Deep Learning Framework | Although the article addresses head and neck cancer and uses AI for segmentation, it is excluded because it does not meet the requirement for direct, numerical comparison of imaging modalities (e.g., PET vs. CT or PET/CT vs. CT). The analysis centers on algorithmic training strategies, not modality performance. |
| de Koster et al., 2022 | Quantitative classification and radiomics of [18F]FDG-PET/CT in indeterminate thyroid nodules | Does not exclusively focus on head and neck malignancies (includes indeterminate/borderline thyroid lesions). Fails to provide a direct, numerical comparison of imaging modalities (PET vs. CT vs. PET/CT) using objective metrics. The analysis centers on radiomic models and SUV thresholds, not modality-specific diagnostic performance. |
| Futsaether et al., | Deep learning GTV segmentation based on PET/CT | Conference Paper/ Inadequate data |
| De Biase et al., 2022 | Slice-by-slice deep learning aided oropharyngeal cancer segmentation on PET and CT images | Although the article addresses head and neck cancer and uses AI/ML for segmentation, it is excluded because it does not meet the requirement for direct, numerical comparison of imaging modalities (e.g., PET vs. CT or PET/CT vs. CT). The analysis focuses on algorithm performance with multi-modal input, not modality-specific comparisons. |
| Bollen et al., 2022 | Automatic delineation of head and neck gross tumor volume using multimodal information | Incomplete Quantitative comparison |
| Fontaine et al., 2022 | Cleaning radiotherapy contours for radiomics studies, is it worth it? A head and neck cancer study | While the article addresses head and neck cancer and uses radiomics, it is excluded because it does not meet two inclusion criteria: AI/ML as a core component (focuses on contour preprocessing, not AI/ML-driven tools). Quantitative comparison of imaging modalities (PET vs. CT vs. PET/CT). The analysis centers on contour accuracy, not modality-specific performance or AI/ML methodology. |
| Tang et al., | Diagnosis of lymph node metastasis in head and neck squamous cell carcinoma using deep learning | While the article addresses head and neck cancer and employs AI/ML for metastasis diagnosis, it is excluded because it does not involve PET/CT, CT, or PET imaging. The study relies on histopathology (HE-stained slides), which falls outside the scope of the systematic review’s focus on PET-based AI applications. |
| Savjani et al., 2021 | Head and Neck Oropharyngeal GTV Autosegmentation: Combining nnU-Net With Shape Representation Loss Driven by a Variational Autoencoder Model | Conference Paper/ Inadequate data |
| Shiri et al., 2021 | Fully Automated Gross Tumor Volume Delineation From PET in Head and Neck Cancer Using Deep Learning Algorithms | Although the article addresses head and neck cancer and employs advanced AI for PET-based segmentation, it does not meet the requirement for direct, numerical comparison of imaging modalities (e.g., PET vs. CT or PET/CT vs. PET). The analysis centers on AI methodology, not modality performance. Thus, it is excluded. |
| Huynh et al., | Tuning deep learning models for automatic segmentation of head and neck cancers in PET/CT images | Although the article addresses head and neck cancer and employs AI for segmentation, it is excluded because it does not meet the requirement for direct, numerical comparison of imaging modalities (e.g., PET vs. CT or PET/CT vs. CT). The analysis focuses on AI architecture optimization rather than modality-specific diagnostic or segmentation performance. |
| Theljani et al., | Contribution of PET and CT Images to Machine Learning for Contouring GTVs in Head and Neck Cancer | Because it does not provide a direct, quantitative head-to-head comparison of at least two specified imaging modalities on segmentation performance, it should be Excluded. |
| Ren et al., | End-to-end head & neck tumor auto-segmentation using CT/PET and MRI without deformable registration | Conference Paper/ Inadequate data |
| Chen et al., 2021 | Attention Guided Lymph Node Malignancy Prediction in Head and Neck Cancer | While the article addresses head and neck cancer and uses advanced AI/ML for LN malignancy prediction, it is excluded because it does not meet the requirement for direct, numerical comparison of imaging modalities (e.g., PET vs. CT or PET/CT vs. CT). The analysis focuses on algorithmic improvements rather than modality-specific diagnostic performance. |
| Yousefirizi et al., 2021 | Consolidating deep learning framework with active contour model for improved PET-CT segmentation | While the article addresses head and neck cancer and employs advanced AI/ML for segmentation, it is excluded because it does not meet the requirement for direct, numerical comparison of imaging modalities (e.g., PET vs. CT or PET/CT vs. CT). The analysis evaluates algorithmic strategies, not modality-specific diagnostic or segmentation performance. |
| Shiri et al., 2021 | Fully automated head and neck malignant lesions segmentation using multimodality PET/CT imaging and a deep convolutional network | Conference Paper/ Inadequate data |
| Hirata et al., 2021 | A Preliminary Study to Use SUVmax of FDG PET-CT as an Identifier of Lesion for Artificial Intelligence | Does not explicitly focus on head and neck cancer. Does not involve AI/ML as a core methodology (focuses on SUVmax validation, not AI algorithms). Lacks comparisons of imaging modalities (PET, CT, PET/CT) for diagnostic or segmentation performance. |
| Han et al., 2021 | Genetic alterations associated with 18F-fluorodeoxyglucose positron emission tomography/computed tomography in head and neck squamous cell carcinoma | Does not use AI/ML as a core component for diagnosis, segmentation, or prognosis (elastic-net regression is ancillary to genetic analysis). Fails to provide a quantitative comparison of imaging modalities (PET, CT, or PET/CT) for diagnostic or segmentation performance. |
| Lin et al., 2021 | Application of Pet-CT Fusion Deep Learning Imaging in Precise Radiotherapy of Thyroid Cancer | Lacks AI/ML-driven methodology (despite the title’s reference to deep learning, the abstract does not implement or test AI/ML models). Fails to provide a direct, numerical comparison of imaging modalities (PET, CT, or PET/CT) as required. |
| Haider et al., 2020 | PET/CT radiomics signature of human papilloma virus association in oropharyngeal squamous cell carcinoma | it fails to provide the required quantitative head-to-head comparison of PET, CT, and/or PET/CT on the same diagnostic or segmentation outcome and is therefore excluded. |
| Yang et al., 2020 | Extracting and Selecting Robust Radiomic Features from PET/MR Images in Nasopharyngeal Carcinoma | Does not employ AI/ML methodologies (relies on statistical analysis, not AI-driven tools). Uses PET/MR instead of the specified PET/CT, CT, or PET modalities, and does not compare these modalities. |
| Olin et al., 2020 | Feasibility of Multiparametric Positron Emission Tomography/Magnetic Resonance Imaging as a One-Stop Shop for Radiation Therapy Planning for Patients with Head and Neck Cancer | Focuses on PET/MRI (not PET/CT, CT, or PET) for radiotherapy planning. Does not provide a quantitative comparison of PET, CT, or PET/CT for diagnosis, segmentation, or prognosis. |
| Dohopolski et al., 2020 | Predicting lymph node metastasis in patients with oropharyngeal cancer by using a convolutional neural network with associated epistemic and aleatoric uncertainty | While the article addresses head and neck cancer and employs advanced AI/ML techniques, it is excluded because it does not meet the requirement for direct, numerical comparison of imaging modalities (e.g., PET vs. CT or PET/CT vs. CT). The analysis focuses on model reliability and uncertainty, not modality-specific performance. |
| Chen et al., 2020 | Segmentation Guided Classification Scheme for Lymph Node Malignancy Prediction in Head and Neck Cancer | Conference Paper/ Inadequate data |
| Thomas et al., 2020 | Use of Machine Learning to Differentiate Residual Tumor from Radiation Changes in Head and Neck Cancer Patients Treated with Definitive Chemoradiotherapy | Conference Paper/ Inadequate data |
| Du et al., 2020 | Machine Learning Methods for Optimal Radiomics-Based Differentiation Between Recurrence and Inflammation: Application to Nasopharyngeal Carcinoma Post-therapy PET/CT Images | While the article addresses head and neck cancer and employs AI/ML for recurrence vs. inflammation classification, it is excluded because it does not meet the requirement for direct, numerical comparison of imaging modalities (e.g., PET vs. CT or PET/CT vs. CT). The analysis is confined to PET-derived radiomics within a PET/CT framework, not modality-specific diagnostic performance. |
| Comelli et al., 2020 | Development of a new fully three-dimensional methodology for tumours delineation in functional images | Does not focus exclusively on head and neck malignancies. Lacks AI/ML as a core methodology (uses traditional active contours). Fails to compare PET, CT, or PET/CT for diagnostic/segmentation performance. |
| Dohopolski et al., 2019 | Predicting Lymph Node Metastasis in Patients with Oropharyngeal Cancer by Convolutional Neural Networks with associated Epistemic Uncertainty | Conference Paper/ Inadequate data |
| Chang et al., 2019 | PET Radiotherapy Response Assessment Using Encoder-Decoder Convolutional Neural Network and Pre-treatment Information: A Feasibility of Oropharynx Cancer IMRT | Conference Paper/ Inadequate data |
| Liao et al., 2019 | Radiomics features analysis of PET images in oropharyngeal and hypopharyngeal cancer | AI/ML as a core methodology (no machine learning models for prediction/segmentation). Direct comparison of imaging modalities (PET, CT, or PET/CT). |
| Gao et al., 2019 | Automatic detection of highuptake lesions in oncologic FDG PET using faster RCNN deep learning model | It does not exclusively or primarily focus on head and neck malignancies. It lacks a direct, numerical comparison of imaging modalities (PET/CT, CT, or PET) as required by the inclusion criteria. |
| Amyar et al., 2019 | Contribution of class activation map on WB PET deep features for primary tumour classification | It does not focus exclusively or primarily on head and neck malignancies (analyzes multiple cancer types). It lacks direct, numerical comparisons of PET, CT, or PET/CT as standalone or combined modalities. |
| Rosvoll Groendahl et al., 2019 | Comparison of automatic tumour segmentation approaches for head and neck cancers in PET/CT images | Conference Paper/ Inadequate data |
| Gouw et al., 2019 | Predicting midtreatment FDG PET in head and neck cancer | While the article addresses head and neck cancer and employs AI/ML (neural network) for PET prediction, it does not meet the requirement for direct, numerical comparison of imaging modalities (e.g., PET vs. CT or PET/CT vs. CT). The analysis centers on midtreatment PET prediction accuracy, not modality-specific performance. Thus, it is excluded. |
| Russo et al., 2019 | A machine learning segmentation approach for the extraction of radiomic features in PET studies | While the article addresses head and neck cancer and employs AI/ML for PET segmentation, it is excluded because it does not meet the requirement for direct, numerical comparison of imaging modalities (e.g., PET vs. CT or PET/CT vs. CT). The study evaluates PET-only segmentation accuracy, not modality-specific diagnostic or prognostic performance. |
| Chen et al., 2019 | Combining many-objective radiomics and 3D convolutional neural network through evidential reasoning to predict lymph node metastasis in head and neck cancer | While the article addresses head and neck cancer and employs advanced AI/ML for LNM prediction, it is excluded because it does not meet the requirement for direct, numerical comparison of imaging modalities (e.g., PET vs. CT or PET/CT vs. CT). The analysis evaluates methodological synergy (radiomics + CNN) rather than modality-specific diagnostic performance. |
| Comelli et al., 2019 | Active contour algorithm with discriminant analysis for delineating tumors in positron emission tomography | Does not focus exclusively on head and neck malignancies. Lacks AI/ML as a core methodology (uses traditional active contours). Fails to compare PET, CT, or PET/CT for diagnostic/segmentation performance. |
| Zhou et al., 2018 | Predicting Lymph Node Metastasis in Head and Neck Cancer by Combining Many-objective Radiomics and 3-dimensioal Convolutional Neural Network through Evidential Reasoning | Conference Paper/ Inadequate data |
| Du et al., 2018 | Machine learning methods for optimal differentiation of recurrence versus inflammation from post-therapy nasopharyngeal18 F-FDG PET/CT images | meeting report/ Inadequate data |
| Parkinson et al., 2018 | Target volume delineation of PET post one cycle of induction chemotherapy in oropharyngeal cancer | Conference Paper/ Inadequate data |
| Huang et al., 2018 | Fully Automated Delineation of Gross Tumor Volume for Head and Neck Cancer on PET-CT Using Deep Learning: A Dual-Center Study | While the article addresses head and neck cancer and employs AI/ML (DCNN) for tumor segmentation, it is excluded because it does not meet the requirement for direct, numerical comparison of imaging modalities (e.g., PET vs. CT or PET/CT vs. CT). The analysis evaluates algorithm accuracy against a manual gold standard, not modality-specific diagnostic or segmentation performance. |
| Li et al., 2018 | Use of radiomics combined with machine learning method in the recurrence patterns after intensity-modulated radiotherapy for nasopharyngeal carcinoma: A preliminary study | While the article addresses head and neck cancer (NPC) and employs AI/ML for recurrence prediction, it is excluded because it does not meet the requirement for direct, numerical comparison of PET/CT, CT, or PET. The analysis is confined to MRI-derived radiomics, which falls outside the scope of the systematic review’s focus on PET/CT, CT, or PET applications. |
| Berthon et al., 2017 | Head and neck target delineation using a novel PET automatic segmentation algorithm | While the article addresses head and neck cancer and employs AI/ML (ATLAAS) for PET segmentation, it is excluded because it does not meet the requirement for direct, numerical comparison of imaging modalities (e.g., PET vs. CT or PET/CT vs. CT). The analysis focuses on algorithm validation against a CT/MRI reference, not modality-specific diagnostic or segmentation performance. |
| Wang et al., 2016 | Can parameters other than minimal axial diameter in MRI and PET/CT further improve diagnostic accuracy for equivocal retropharyngeal lymph nodes in nasopharyngeal carcinoma? | While the article addresses head and neck cancer (NPC) and employs AI/ML (neural network) for nodal diagnosis, it is excluded because it does not meet the requirement for direct, numerical comparison of PET/CT, CT, or PET. The analysis combines MRI and PET/CT parameters but does not evaluate the diagnostic performance of PET/CT, CT, or PET as standalone modalities. |
| Wang et al., 2016 | Can parameters other than minimal axial diameter in MRI and PET/CT further improve the diagnostic accuracy of equivocal retropharyngeal lymph nodes in nasopharyngeal carcinoma? | While the article addresses head and neck cancer (NPC) and employs AI/ML (neural network) for nodal diagnosis, it is excluded because it does not meet the requirement for direct, numerical comparison of PET/CT, CT, or PET. The analysis combines MRI and PET/CT parameters but does not evaluate the diagnostic performance of PET/CT, CT, or PET as standalone modalities. |
| Zhuang et al., 2016 | Generic and robust method for automatic segmentation of PET images using an active contour model | Does not focus exclusively or primarily on head and neck malignancies. Lacks AI/ML-driven methodology. Fails to compare PET/CT, CT, or PET as standalone or combined modalities. |
| Wu et al., 2012 | Automatic detection and classification of nasopharyngeal carcinoma on PET/CT with support vector machine | While the article addresses head and neck cancer (NPC) and employs AI/ML (SVM), it is excluded because it does not meet the requirement for direct, numerical comparison of imaging modalities (e.g., PET vs. CT or PET/CT vs. CT). The study focuses on combined PET/CT features rather than modality-specific diagnostic or segmentation performance. |
| Lei et al., | Biological gross tumour volume (GTVB) in head and neck cancer (HNC): Comparison of automated segmentation tools | Does not employ AI/ML as a core methodology (FLAB segmentation is not clearly an AI/ML technique). Fails to provide direct, numerical comparisons of PET, CT, or PET/CT as standalone modalities. The analysis is confined to algorithmic segmentation on PET/CT, not modality-specific performance. |
| Yu et al., 2008 | Coregistered FDG PET/CT-based textural characterization of head and neck cancer for radiation treatment planning. | While the article addresses head and neck cancer and employs AI/ML (KNN/DT classifiers), it is excluded because it does not meet the requirement for direct, numerical comparison of imaging modalities (e.g., PET vs. CT or PET/CT vs. CT). The analysis focuses on textural feature discrimination using combined PET/CT data, not modality-specific performance. |
| Kann et al., 2018 | Pretreatment identification of head and neck cancer nodal metastasis and extranodal extension using deep learning neural networks | While the AI model is compared to human clinician performance (using metrics like AUC, sensitivity, specificity), there is no comparison between PET/CT, CT, or PET. |
| Zhong et al., 2022 | Tumor radiomics signature for artificial neural network-assisted detection of neck metastasis in patient with tongue cancer | because it fails to provide a direct, quantitative comparison of at least two specified imaging modalities (PET/CT, CT, or PET) as required by the inclusion rules. |
| Heydarheydari et al., 2023 | Auto-segmentation of head and neck tumors in positron emission tomography images using non-local means and morphological frameworks | It does not use AI/ML as a core component (relies on traditional image processing). It does not compare multiple imaging modalities (e.g., PET vs. CT or PET/CT) quantitatively. |
| Cardenas et al., 2021 | Generating high-quality lymph node clinical target volumes for head and neck cancer radiation therapy using a fully automated deep learning-based approach | fails to compare at least two of the specified imaging modalities (PET/CT, CT, PET). The study exclusively relies on CT scans without incorporating or contrasting PET imaging, which is a requirement for inclusion in the systematic review. |
| Bianconi et al., 2023 | Performance Analysis of Six Semi-Automated Tumour Delineation Methods on [18F] Fluorodeoxyglucose Positron Emission Tomography/Computed Tomography (FDG PET/CT) in Patients with Head and Neck Cancer | No AI/ML is used (only manual or semi-automated thresholding/algorithms). No comparison of imaging modalities (PET/CT vs. CT or PET). |
| Bagci et al., 2013 | Predicting future morphological changes of lesions from radiotracer uptake in 18F-FDG-PET images | Does not focus on head and neck cancer. Uses non-AI/ML methods (graph-based segmentation and feature extraction without ML training/validation). Does not compare imaging modalities (e.g., PET vs. CT). |
| Chen et al., 2019 | Automatic PET cervical tumor segmentation by combining deep learning and anatomic prior | It focuses on cervical cancer, not head and neck malignancies. It does not compare imaging modalities (e.g., PET vs. CT or PET/CT). |
| Comelli et al., 2020 | Fully 3D Active Surface with Machine Learning for PET Image Segmentation | fails to quantitatively compare at least two imaging modalities (e.g., PET/CT, CT, or PET). The comparison is methodological (3D vs. 2D segmentation techniques on PET alone), not inter-modality. |
| Hatt et al., 2009 | A fuzzy locally adaptive Bayesian segmentation approach for volume determination in PET | Does not focus on head and neck cancer. Uses non-AI/ML methods (statistical fuzzy Bayesian segmentation). Does not compare imaging modalities (e.g., PET vs. CT or PET/CT). |
| Kawauchi et al., 2020 | A convolutional neural network-based system to classify patients using FDG PET/CT examinations | it does not compare imaging modalities (e.g., PET/CT vs. CT or PET). The analysis focuses on AI classification accuracy against physician labels, not modality performance. Thus, it is excluded from the systematic review. |
| Liu et al., 2024 | Radiomics-based machine learning models for differentiating pathological subtypes in cervical cancer: a multicenter study | The article is excluded because it does not focus on head and neck malignancies, despite meeting the other criteria (AI/ML use and multi-modality comparison). The systematic review scope is limited to head and neck cancers, and cervical cancer falls outside this domain. |
| Naser et al., 2020 | Tumor Segmentation in Patients with Head and Neck Cancers Using Deep Learning Based-on Multi-modality PET/CT Images | Conference Paper/ Inadequate data |
| Naser et al., 2021 | Head and Neck Cancer Primary Tumor Auto Segmentation Using Model Ensembling of Deep Learning in PET/CT Images | No direct, quantitative comparison of PET/CT with CT or PET alone. Focus is on algorithmic improvements (ResUnet + ensembling), not inter-modality performance. |
| Nishigaki et al., 2024 | Vision transformer to differentiate between benign and malignant slices in (18)F-FDG PET/CT | It does not focus on head and neck cancer (general oncological application). It lacks a quantitative comparison of imaging modalities (e.g., PET vs. CT). |
| Yeh et al., 2021 | Predicting aggressive histopathological features in esophageal cancer with positron emission tomography using a deep convolutional neural network | It does not focus on head and neck cancer (targets esophageal cancer). It lacks a quantitative comparison of imaging modalities (e.g., PET vs. CT or PET/CT). |
| Zhou et al., 2020 | Quantitative PET Imaging and Clinical Parameters as Predictive Factors for Patients With Cervical Carcinoma: Implications of a Prediction Model Generated Using Multi-Objective Support Vector Machine Learning | It focuses on cervical cancer, outside the scope of head and neck malignancies. It does not compare imaging modalities (e.g., PET vs. CT or PET/CT). |
| Li et al., 2024 | SwinCross: Cross-modal Swin transformer for head-and-neck tumor segmentation in PET/CT images | Focuses on head and neck cancer. Uses AI/ML (SwinCross Transformer) as a core component. Provides quantitative comparison of PET and CT through cross-modal analysis, emphasizing their combined utility. |
| Gruteser et al., 2022 | Poster: Head and Neck Tumor Segmentation With Sliced 3D PET Scans | Conference Paper/ Inadequate data |
| Salahuddin et al., 2022 | HNT-AI: An Automatic Segmentation Framework for Head and Neck Primary Tumors and Lymph Nodes in FDG- PET/CT Images | Conference Paper/ Inadequate data |
| Murugesan et al., 2021 | Head and Neck Primary Tumor Segmentation Using Deep Neural Networks and Adaptive Ensembling | Conference Paper/ Inadequate data |
| Andrearczyk et al., 2021 | Multi-task Deep Segmentation and Radiomics for Automatic Prognosis in Head and Neck Cancer | Conference Paper/ Inadequate data |
| Hung et al., 2022 | Future Trends of PET/MR and Utility of AI in Multi-Modal Imaging | It does not focus on head and neck cancer as a primary subject. It lacks core AI/ML methodology (no model training/validation). It does not quantitatively compare imaging modalities (e.g., PET vs. CT or PET/CT) for head and neck cancer diagnosis. |
| [Oreiller](https://scholar.google.com/citations?user=DtxIr6wAAAAJ&hl=en&oi=sra) et al., 2022 | 1st 3D Head and Neck Tumor Segmentation in PET/CT Challenge, HECKTOR 2020, which was held in conjunction with 23rd International Conference on Medical Image Computing and Computer-Assisted Intervention, MICCAI 2020 | The article (proceedings overview) meets the first two inclusion criteria but fails to provide a direct, quantitative comparison of imaging modalities (e.g., PET vs. CT). The challenge evaluates AI methods for segmentation on combined PET/CT data without isolating modality-specific contributions. Thus, it is excluded from the systematic review. |
| Kao et al., 2001 | Comparing 18-fluoro-2-deoxyglucose positron emission tomography with a combination of technetium 99m tetrofosmin single photon emission computed tomography and computed tomography to detect recurrent or persistent nasopharyngeal carcinomas after radiotherapy | Absence of AI/ML in the diagnostic process. Reliance on traditional imaging analysis without machine learning or deep learning methodologies. |
| Oe et al., 2007 | Detection of local residual tumor after laryngeal cancer treatment using FDG-PET | No AI/ML is used; the methodology is based on conventional SUV thresholding. No comparison of imaging modalities (e.g., PET vs. CT). |
| Nakajo et al., 2025 | Applying deep learning-based ensemble model to [18F]-FDG-PET-radiomic features for differentiating benign from malignant parotid gland diseases | Lack of inter-modality comparison (e.g., PET vs. CT) as required by the systematic review criteria. Thus, the article is excluded from the review. |
| Yang et al., 2023 | Computer-aided diagnostic models to classify lymph node metastasis and lymphoma involvement in enlarged cervical lymph nodes using PET/CT | Focuses on head and neck malignancies (cervical lymph nodes). Uses AI/ML (DL-CNN, SVM) as a core component. Provides quantitative comparison of PET, CT, and PET/CT imaging modalities for diagnostic performance. |
| Mahdi et al., 2024 | Segmentation of Head and Neck Tumors Using Dual PET/CT Imaging: Comparative Analysis of 2D, 2.5D, and 3D Approaches Using UNet Transformer | Lack of direct comparison between PET, CT, or PET/CT as required by the systematic review criteria. Thus, the article is excluded from the review. |
| Comelli et al., 2019 | K-nearest neighbor driving active contours to delineate biological tumor volumes | The methodology prioritizes traditional image processing (active contours) over AI/ML-driven diagnosis. Lack of inter-modality comparison (PET vs. CT or PET/CT) as required. Thus, the article is excluded from the systematic review. |

Supplementary 2-Excluded studies with the reasons for exclusion

| **Item** | **Dong 2024** | **Groendahl 2021** | **Guo 2020** | **Huang 2022 (ISA)** | **Huang 2022 (TG)** | **Mahdi 2024** | **Moe 2021** | **Oreiller 2022** | **Shiri 2024** | **Zhao 2019** | **Zhao 2024 (MMCA)** |
| --- | --- | --- | --- | --- | --- | --- | --- | --- | --- | --- | --- |
| **C1.1** | Yes | Yes | Yes | Yes | Yes | Yes | Yes | Yes | Yes | Yes | Yes |
| **C1.2** | Yes | Yes | Yes | Yes | Yes | Yes | Yes | Yes | Yes | Yes | Yes |
| **C1.3** | NA | NA | NA | NA | NA | NA | NA | NA | NA | NA | NA |
| **C1.4** | NA | NA | NA | NA | NA | NA | NA | NA | NA | NA | NA |
| **C1 ROB** | **Low** | **Low** | **Low** | **Low** | **Low** | **Low** | **Low** | **Low** | **Low** | **Low** | **Low** |
| **C2.1** | Yes | Yes | Yes | Yes | Yes | Yes | Yes | Yes | Yes | Yes | Yes |
| **C2.2** | Yes | Yes | Yes | Yes | Yes | Yes | Yes | Yes | Yes | Yes | Yes |
| **C2.3** | Yes | Yes | Yes | Yes | Yes | Yes | Yes | Yes | Yes | Yes | Yes |
| **C2.4** | Yes | Yes | Yes | Yes | Yes | Yes | Yes | Yes | Yes | Yes | Yes |
| **C2 ROB** | **Low** | **Low** | **Low** | **Low** | **Low** | **Low** | **Low** | **Low** | **Low** | **Low** | **Low** |
| **C3.1** | Yes | Yes | Yes | Yes | Yes | Yes | Yes | Yes | Yes | Yes | Yes |
| **C3.2** | Yes | Yes | Yes | Yes | Yes | Yes | Yes | Yes | Yes | Yes | Yes |
| **C3 ROB** | **Low** | **Low** | **Low** | **Low** | **Low** | **Low** | **Low** | **Low** | **Low** | **Low** | **Low** |
| **C4.1** | Yes | Yes | Yes | Yes | Yes | Yes | Yes | Yes | Yes | Yes | Yes |
| **C4.2** | Yes | Yes | Yes | Yes | Yes | Yes | Yes | Yes | Yes | Yes | Yes |
| **C4.3** | Yes | Yes | Yes | Yes | Yes | Yes | Yes | Yes | Yes | Yes | Yes |
| **C4.4** | Yes | Yes | Yes | Yes | Yes | Yes | Yes | Yes | Yes | Yes | Yes |
| **C4 ROB** | **Low** | **Low** | **Low** | **Low** | **Low** | **Low** | **Low** | **Low** | **Low** | **Low** | **Low** |
| **Overall ROB** | **Low** | **Low** | **Low** | **Low** | **Low** | **Low** | **Low** | **Low** | **Low** | **Low** | **Low** |

Supplementary 3-Risk of Bias (ROB) assessment of the included studies by QUADAS-C

|  | **Dong 2024** | **Groendahl 2021** | **Guo 2020** | **Huang 2022 (ISANet)** | **Huang 2022 (TGNet)** | **Mahdi 2024** | **Moe 2021** | **Oreiller 2022** | **Shiri 2024** | **Zhao 2019** | **Zhao 2024** |
| --- | --- | --- | --- | --- | --- | --- | --- | --- | --- | --- | --- |
| **Item 1** | 1 | 1 | 1 | 1 | 1 | 1 | 1 | 1 | 1 | 1 | 1 |
| **Item 2** | 1 | 1 | 1 | 1 | 1 | 1 | 1 | 1 | 1 | 1 | 1 |
| **Item 3** | 1 | 1 | 1 | 1 | 1 | 1 | 1 | 1 | 1 | 1 | 1 |
| **Item 4** | 1 | 1 | 1 | 1 | 1 | 1 | 1 | 1 | 1 | 1 | 1 |
| **Item 5** | 0 | 0 | 0 | 0 | 0 | 0 | 1 | 1 | 1 | 1 | 1 |
| **Item 6** | 1 | 1 | 1 | 1 | 1 | 1 | 1 | 1 | 1 | 1 | 1 |
| **Item 7** | 1 | 1 | 1 | 1 | 1 | 1 | 1 | 1 | 1 | 1 | 1 |
| **Item 8** | 1 | 1 | 1 | 1 | 1 | 1 | 1 | 1 | 1 | 1 | 1 |
| **Item 9** | 1 | 1 | 1 | 1 | 1 | 1 | 1 | 1 | 1 | 1 | 1 |
| **Item 10** | 1 | 1 | 1 | 1 | 1 | 1 | 1 | 1 | 1 | 1 | 1 |
| **Item 11** | 0 | 0 | 0 | 0 | 0 | 0 | 0 | 0 | 0 | 0 | 0 |
| **Item 12** | 0 | 1 | 0 | 0 | 0 | 0 | 0 | 1 | 0 | 0 | 0 |
| **Item 13** | 0 | 0 | 0 | 0 | 0 | 0 | 0 | 0 | 0 | 0 | 0 |
| **Item 14** | 1 | 1 | 1 | 1 | 1 | 1 | 1 | 1 | 1 | 1 | 1 |
| **Item 15** | 1 | 1 | 1 | 1 | 1 | 1 | 1 | 1 | 1 | 1 | 1 |
| **Item 16** | 1 | 1 | 1 | 1 | 1 | 1 | 1 | 1 | 1 | 1 | 1 |
| **Item 17** | 1 | 1 | 1 | 1 | 1 | 1 | 1 | 1 | 1 | 1 | 1 |
| **Item 18** | 0 | 0 | 0 | 0 | 0 | 0 | 0 | 1 | 0 | 0 | 0 |
| **Item 19** | 1 | 0 | 1 | 1 | 1 | 1 | 1 | 0 | 1 | 1 | 1 |
| **Item 20** | 1 | 1 | 1 | 1 | 1 | 1 | 1 | 1 | 1 | 1 | 1 |
| **Item 21** | 1 | 1 | 1 | 1 | 1 | 1 | 1 | 1 | 1 | 1 | 1 |
| **Item 22** | 1 | 1 | 1 | 1 | 1 | 1 | 1 | 1 | 1 | 1 | 1 |
| **Item 23** | 1 | 1 | 1 | 1 | 1 | 1 | 1 | 1 | 1 | 1 | 1 |
| **Item 24** | 1 | 1 | 1 | 1 | 1 | 1 | 1 | 1 | 1 | 1 | 1 |
| **Item 25** | 1 | 1 | 1 | 1 | 1 | 1 | 1 | 1 | 1 | 1 | 1 |
| **Item 26** | 1 | 1 | 1 | 1 | 1 | 1 | 1 | 1 | 1 | 1 | 1 |
| **Item 27** | 0 | 0 | 0 | 0 | 0 | 0 | 0 | 1 | 1 | 0 | 0 |
| **Item 28** | 1 | 1 | 1 | 1 | 1 | 1 | 1 | 1 | 1 | 1 | 1 |
| **Item 29** | 1 | 1 | 1 | 1 | 1 | 1 | 1 | 1 | 1 | 1 | 1 |
| **Item 30** | 1 | 0 | 1 | 1 | 1 | 1 | 1 | 1 | 1 | 1 | 1 |
| **Item 31** | 0 | 0 | 0 | 0 | 0 | 0 | 0 | 0 | 0 | 0 | 1 |
| **Item 32** | 0 | 0 | 1 | 1 | 0 | 1 | 0 | 0 | 1 | 0 | 1 |
| **Item 33** | 1 | 1 | 1 | 1 | 1 | 1 | 1 | 1 | 1 | 1 | 1 |
| **Item 34** | 1 | 1 | 1 | 1 | 1 | 1 | 1 | 1 | 1 | 1 | 1 |
| **Item 35** | 1 | 1 | 1 | 1 | 1 | 1 | 1 | 1 | 1 | 1 | 1 |
| **Item 36** | 1 | 0 | 1 | 1 | 1 | 1 | 1 | 1 | 1 | 1 | 1 |
| **Item 37** | 0 | 1 | 1 | 1 | 1 | 1 | 1 | 1 | 1 | 1 | 1 |
| **Item 38** | 1 | 1 | 1 | 1 | 1 | 1 | 1 | 1 | 1 | 1 | 1 |
| **Item 39** | 1 | 1 | 1 | 1 | 1 | 1 | 1 | 1 | 1 | 1 | 1 |
| **Item 40** | 0 | 0 | 0 | 0 | 0 | 0 | 0 | 0 | 0 | 0 | 0 |
| **Item 41** | 0 | 0 | 0 | 0 | 0 | 0 | 0 | 0 | 0 | 0 | 0 |
| **Item 42** | 1 | 1 | 1 | 1 | 1 | 1 | 1 | 1 | 1 | 1 | 1 |
| **Total** | **31** | **30** | **33** | **33** | **32** | **33** | **33** | **34** | **35** | **33** | **35** |

Supplementary 4-Item-wise CLAIM Assessment Across All Included Studies
